# Current AI technologies in cancer diagnostics and treatment

**DOI:** 10.1186/s12943-025-02369-9

**Published:** 2025-06-02

**Authors:** Ashutosh Tiwari, Soumya Mishra, Tsung-Rong Kuo

**Affiliations:** 1 International Ph.D. Program in Biomedical Engineering, College of Biomedical Engineering, Taipei Medical University, Taipei, 11031 Taiwan; 2 Capacity Development Fellow, BRICS Research Institute, Durban University of Technology, Durban, 4001 South Africa; 3 Department of Biotechnology, School of Interdisciplinary Education and Research, Guru Ghasidas Vishwavidyalaya, Bilaspur, Chhattisgarh 495001 India; 4 Graduate Institute of Nanomedicine and Medical Engineering, College of Biomedical Engineering, Taipei Medical University, Taipei, 11031 Taiwan; 5 Precision Medicine and Translational Cancer Research Center, Taipei Medical University Hospital, Taipei, 11031 Taiwan

**Keywords:** Cancer, Artificial intelligence (AI), Machine learning (ML), Cancer diagnosis, Deep learning (DL), Precision oncology

## Abstract

Cancer continues to be a significant international health issue, which demands the invention of new methods for early detection, precise diagnoses, and personalized treatments. Artificial intelligence (AI) has rapidly become a groundbreaking component in the modern era of oncology, offering sophisticated tools across the range of cancer care. In this review, we performed a systematic survey of the current status of AI technologies used for cancer diagnoses and therapeutic approaches. We discuss AI-facilitated imaging diagnostics using a range of modalities such as computed tomography, magnetic resonance imaging, positron emission tomography, ultrasound, and digital pathology, highlighting the growing role of deep learning in detecting early-stage cancers. We also explore applications of AI in genomics and biomarker discovery, liquid biopsies, and non-invasive diagnoses. In therapeutic interventions, AI-based clinical decision support systems, individualized treatment planning, and AI-facilitated drug discovery are transforming precision cancer therapies. The review also evaluates the effects of AI on radiation therapy, robotic surgery, and patient management, including survival predictions, remote monitoring, and AI-facilitated clinical trials. Finally, we discuss important challenges such as data privacy, interpretability, and regulatory issues, and recommend future directions that involve the use of federated learning, synthetic biology, and quantum-boosted AI. This review highlights the groundbreaking potential of AI to revolutionize cancer care by making diagnostics, treatments, and patient management more precise, efficient, and personalized.

**Graphical Abstract:**

This graphical abstract schematically illustrates the progressive role of artificial intelligence in the cancer treatment continuum.

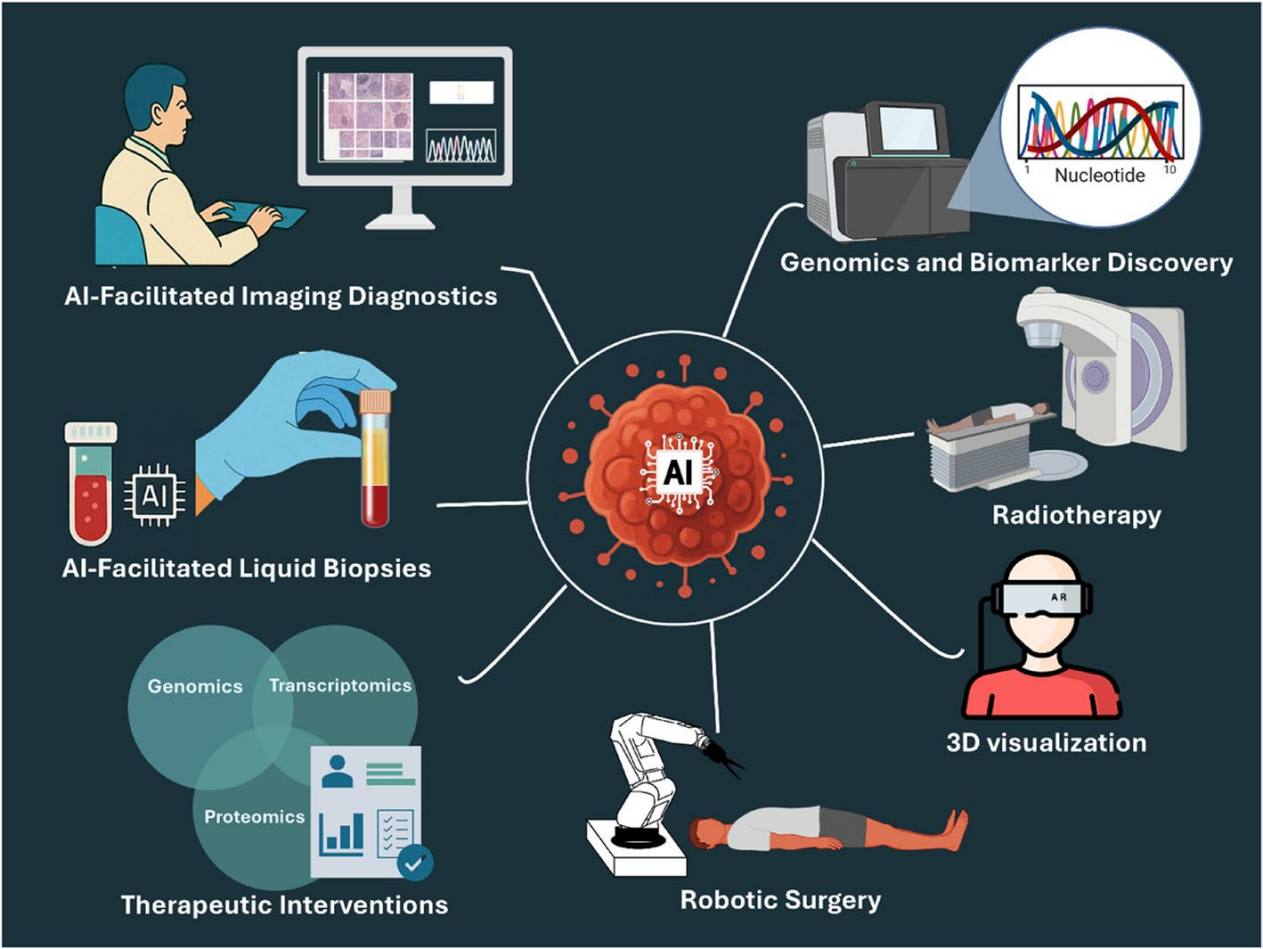

## Introduction

Cancer, an illness that can affect people from all walks of life, is an intricate worldwide health concern that continues to require attention. Cancer is a disease that affects people regardless of age and causes suffering all around the world. Cancer is the second most prevalent cause of mortality worldwide, accounting for one in six deaths in 2020, according to the World Health Organization (WHO) [1]. Through a gradual accumulation of biological and therapeutic knowledge which accelerated with the development of molecular-cell biology and genetics in the second half of the twentieth century, modern medicine altered that perspective. Together with more-recent technological developments, this progress has made it possible to comprehend the disease in ways that were never possible before. The term "cancer" now encompasses hundreds of different kinds of diseases with similar basic characteristics. Beyond figuring out a particular cancer type's genetic fingerprint and molecular composition, we now know how crucial the systemic and local tumor environment is to the disease's progression and presentation. In recent years, interactions between the immune system and the immunological tumor microenvironment (TME) has particularly garnered notice [2, 3].

### The role of artificial intelligence (AI) in modern oncology

AI refers to the wide area of computer science where algorithms or machines are designed to mimic human intellect. In machine learning (ML), a subfield of AI, computers carry out predetermined tasks and use statistical techniques to find hidden patterns in data and enhance model performance [4]. Unlike standard ML, the ML subfield of deep learning (DL) does not rely on human-defined heuristics to complete a task. Instead, DL uses the capability of multilayered neural networks to eliminate manual feature extraction labor and allow for the self-discovery of features that humans are unaware of or would not have expected [5, 6]. The major AI concepts are listed in Table 1. Electronic health record (HER) clinical notes, diagnostic and procedural reports, and other unstructured data are transformed into discrete data elements using natural language processing (NLP) [7], an adjacent specialization within AI that aims to bridge human language with machine interpretation [8]. Recent developments in the field have significantly improved the technology's efficacy, allowing it to be used to automate the gathering and recording of patient outcomes, progression-free survival (PFS), and other tumor features associated with cancer [9]. The construction of intricate databases and tumor registries may be facilitated by such automation, which recursively boosts the strength of generated models. NLP has been used to match clinical trials and detect possible adverse medication reactions, either alone or in conjunction with ML/DL approaches [10–12]. Furthermore, the use of AI for clinical decision-making is thought to improve the likelihood of early disease diagnosis and predictions using high-resolution imaging and new generation sequencing (NGS) methods. Creating sizable datasets and employing specialized bioinformatic tools have also resulted in the introduction of novel biomarkers for diagnosing cancer, the development of novel tailored medications, and the delivery of potential treatment regimens [13].

**Table 1:** Key AI concepts and architectures relevant to cancer diagnostics and research

Category	Concept / Model	Description & Relevance in Oncology	References
Machine Learning (ML)	Supervised Learning	Learn from labeled data to make predictions. Used for classifying tumors, predicting survival, etc	[14]
	Unsupervised Learning	Discovers hidden patterns in unlabeled data; applied in clustering patients or tumor subtypes	
	Semi-supervised Learning	Combines a small amount of labeled data with a large unlabeled dataset, useful in medical imaging with limited annotations	
	Reinforcement Learning	Learns by trial-and-error through feedback. Applied in treatment policy optimization	
	Feature Engineering	The process of selecting or transforming variables to improve ML performance. Crucial for structured EHR and omics data	
Classical ML Models	Support Vector Machines (SVM)	Effective in high-dimensional spaces (e.g., gene expression data) for classification tasks	[15]
	Random Forests (RF)	Ensemble of decision trees; robust against overfitting, used for biomarker prediction and classification	
	Logistic Regression (LR)	Common baseline model for binary classification in survival and risk prediction	
	k-Nearest Neighbors (k-NN)	Instance-based learner; used in similarity-based drug repositioning and subtype classification	
Deep Learning (DL)	Deep Neural Networks (DNNs)	Multilayered feedforward networks for structured data, widely used in survival prediction	[16–29]
	Convolutional Neural Networks (CNNs)	Specialized for image data (CT, MRI, histopathology); extracts spatial hierarchies in features	
	Recurrent Neural Networks (RNNs)	Suited for sequential data (e.g., patient records); models time-dependent health trajectories	
	Long Short-Term Memory (LSTM)	A type of RNN that captures long-range dependencies; applied in EHR and time-series prognosis	
	Gated Recurrent Units (GRUs)	Efficient RNN variant; used in longitudinal cancer data modeling	
	Residual Networks (ResNet)	DL architecture with skip connections; enables deeper networks for accurate image-based classification. Extensively used in digital pathology	
	Vision Transformers (ViT)	Transformer-based models adapted for image analysis; increasingly used for WSI (whole-slide image) classification	
	LongNet	A transformer variant enabling processing of very long sequences (> 32 k tokens); suitable for high-resolution pathology slide and multi-modal data	
	U-Net	A CNN architecture designed for biomedical image segmentation; heavily used in tumor boundary and organ-at-risk contouring	
	EfficientNet	Optimized CNN with excellent performance at low computational cost; used in real-time image analysis and mobile health apps	
	Graph Neural Networks (GNNs)	Models relational data; used for protein–protein interactions, drug-target graphs, and patient similarity networks	
	Autoencoders (AEs)	Unsupervised models for data compression and denoising; used in omics dimensionality reduction	
	Variational Autoencoders (VAEs)	A probabilistic extension of AEs used for generative tasks (e.g., molecule generation)	
	Generative Adversarial Networks (GANs)	Generate realistic synthetic data (e.g., histopathology images, molecules). Applied in data augmentation and simulation	
	Adversarial Autoencoders (AAEs)	Combines GAN and AE for structured representation learning. Used in molecule and feature generation	
Transformers and Attention Models	Transformer	Core architecture using self-attention; enables context-aware modeling. Used in NLP and multi-modal integration in oncology	[30–39]
	BERT / BioBERT / ClinicalBERT	Pre-trained language models fine-tuned on biomedical texts. Applied to EHR, radiology reports, and literature mining	
	GPT / GPT-3 / GPT-4	Autoregressive transformers used for medical Q&A, summarization, and even synthetic data generation	
	T5 / BioT5	Sequence-to-sequence transformers used in molecular-to-text or image-to-report tasks	
	CLIP (Contrastive Language-Image Pretraining)	Joint vision–language model; maps images and text to a shared space. Applied in pathology image captioning and labeling	
Learning Paradigms	Transfer Learning	Fine-tuning pre-trained models on domain-specific data. Useful in small medical datasets	
	Federated Learning	Decentralized training across institutions without data sharing; supports data privacy in multi-center oncology studies	
	Self-supervised Learning	Learns from unlabeled data using pretext tasks. CHIEF and other models use this for pathology image feature extraction	
	Contrastive Learning	Learns representations by comparing similar/dissimilar pairs. Enhances embedding quality for histology and radiomics	
	Multi-task Learning	Simultaneous learning of related tasks. Improves generalization in cancer subtype classification and prognosis	
Evaluation Metrics	AUROC	Measures model’s ability to discriminate between classes; critical in binary cancer detection tasks	[40–45]
	Accuracy, Sensitivity, Specificity	Basic metrics used to assess model performance	
	Precision, Recall, F1-score	Balance false positives and negatives; important in imbalanced cancer datasets	
	Kaplan–Meier, C-index	Used in survival models to evaluate time-to-event predictions	
	Confusion Matrix	Summarizes classification outcomes; visual tool for error analysis	

### Importance of AI in enhancing cancer diagnostics and treatment

**Fig. 1:**
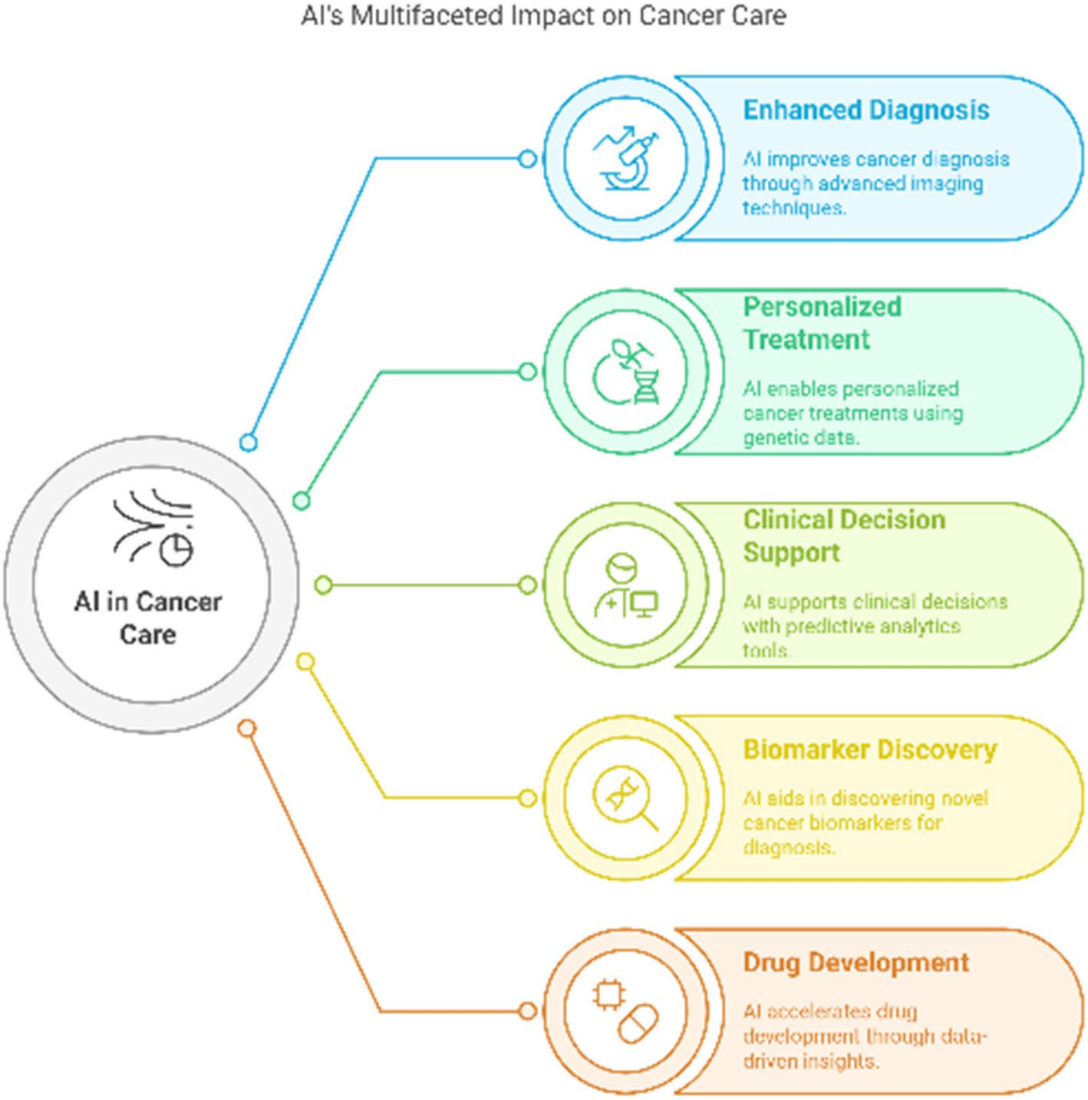
AI's diverse roles in cancer care, including enhanced diagnosis, personalized treatment, clinical decision support, biomarker discovery, and drug development each contributing to improved precision, speed, and outcomes in oncology through data-driven innovations

Numerous studies have suggested that screening can increase early cancer detection and decrease mortality (Fig. 1). However, even in disease groups like breast cancer where screening programs are well-established, discussions about patient selection and risk-benefit trade-offs continue, and concerns have been raised regarding a perceived "one size fits all" approach that is inconsistent with the goals of personalized medicine [46–48]. In the near future, AI algorithms may play a part in enhancing this procedure since they can analyze enormous volumes of multimodal data to find signals that would otherwise be hard to spot [49–51].

### Objectives and scope of this review

This article provides an in-depth review of AI's role in modern cancer diagnostics, consolidating diverse cancer types and AI-facilitated diagnostic approaches into a cohesive overview. AI in oncology enhances diagnosis, treatment, and patient management by increasing precision, efficiency, and personalization. Leveraging ML, DL, and NLP, AI analyzes complex datasets—including pathology reports, clinical records, genomic data, and medical images—to generate insights that support more accurate and timely clinical decisions. Its goals include early detection, personalized treatment planning, and streamlined care delivery to improve patient outcomes. This review spans both research-driven AI innovations and clinical applications, incorporating studies, benchmark models, commercial tools, and regulatory perspectives. It offers valuable insights for a wide audience, including oncologists, AI researchers, informaticians, policymakers, and biomedical engineers. By framing AI as a bridge between predictive and precision oncology, this review supports strategic decision-making and encourages research that translates AI’s theoretical promise into real-world clinical impact.

## AI in cancer diagnostics

AI is developing at an exponential rate. Clinical oncology research is now more focused on comprehending the intricate biological architecture of cancer cell proliferation in order to decipher the molecular origins of cancer. In order to address the current situation of rising cancer mortality rates worldwide, it has also concentrated on processing millions of pertinent cases in big data and computational biology [52]. Furthermore, the use of AI in clinical decision-making is thought to improve the likelihood of early disease diagnoses and predictions using high-resolution imaging and NGS methods. By creating sizable datasets and employing specialized bioinformatic tools, it may also result in introducing novel biomarkers for diagnosing cancer, developing novel tailored medications, and delivering potential treatment regimens [13].

### Imaging-based AI diagnostics

AI, which is based on computational models and bioinformatics-based algorithms, presents medical imaging technology (MIT) with significant opportunities for advancement. It can identify biological alterations and aberrant cellular growth in the body [53]. In addition to being crucial in radiology, AI-assisted MIT has had a significant influence on neuroradiography and medical resonance imaging. Numerous dynamic applications of AI exist, including picture interpretation and categorization, data organization, information storage, information mining, and much more. AI is anticipated to greatly assist pathologists in enhancing diagnostic specificity because of its broad application in biomedical imaging technology [54].

Assessing tumors using traditional radiographic imaging is primarily based on qualitative characteristics, such as tumor density, enhancement patterns, intra-tumoral cellular and acellular compositions (including blood, necrosis, and mineralization), tumor margin regularity, anatomical relationships with surrounding tissues, and impacts on these structures. It is possible to quantify a tumor’s size and shape using one- (1D), two- (2D), and 3-dimensional (3D) analyses. All of these qualitative phenotypic descriptions are referred to as "semantic" traits. In contrast, a quickly developing area known as radiomics is making it possible to digitally decode radiographic pictures into quantitative properties, such as size, shape, and textural pattern descriptors [55]. The automatic quantification of radiographic patterns in medical imaging data has significantly progressed in recent years due to advancements in AI approaches. A subset of AI called DL is particularly promising since it automatically learns feature representations from sample photos and was demonstrated to perform on par with or even better than humans in task-specific applications [5, 56]. DL has shown relative robustness against noise in ground truth labels, among other things, even though it requires enormous datasets for training [57].

In external-beam radiation therapy, tomographic imaging is vital for follow-up care, image guidance, and treatment planning. A CT simulation is typically performed before treatment to image the targeted body part. Using these images, the tumor and nearby critical structures are identified to develop the optimal treatment plan. For tumors near the diaphragm (e.g., liver or lower lung lobe), 4D CT scans may be used to track respiratory motion. MRI is often recommended for brain, paraspinal, head and neck, prostate cancers, and extremity sarcomas due to its superior soft-tissue contrast. MRI scans are fused with CT for tumor delineation and organ-at-risk contouring, or used alone in MRI-only simulations with synthesized CT for planning and dose calculation. Unlike CT and MRI, PET reveals tumor metabolism and helps define dose-escalation volumes, especially in head and neck cancers [58].

AI's automated abilities such as precise tumor volume tracking over time, simultaneous monitoring of multiple lesions, linking phenotypic nuances to genotypes, and predicting outcomes via comparisons with vast tumor databases—can enhance clinicians' qualitative judgment. DL methods further improve generalizability across diseases and imaging types, reduce noise sensitivity and errors, and may enable earlier treatments and significant clinical advances. While most studies remain preclinical, the evolution of automated radiographic "radiomic" markers may ultimately shift cancer diagnostics by identifying actionable tumor abnormalities [59].

Today's digital pathology faces three core challenges that must be addressed as digitization expands, and AI capabilities evolve, These include:

1. improved efficiency, quality control, and image management in laboratory operations;
2. clinical decision support, where algorithms are used to identify areas of interest or make specific diagnoses; and
3. research and development, where new biomarkers [60], transcriptomics [61], and correlations between image characteristics and prognostics have been discovered [62].

The application of AI for digital pathology predates the introduction of whole-slide images (WSIs). Previous research showed that computer vision and AI methods can distinguish between diseases in pathology images. However, previously chosen regions of interest (ROIs) made up the majority of those image datasets. Because pathologists must first choose the areas of interest, this approach is extremely time-consuming and technically impractical to integrate into a laboratory's clinical process [63]. One major obstacle in healthcare systems is the early-stage identification of cancer, mainly because early stages of cancer are modest and frequently asymptomatic. Early cancer detection is essential for effective treatment and higher survival rates, but there are a number of reasons that make this process complex and challenging. This investigation explores the complexities of these problems, including systemic, technological, and biological ones, and emphasizes how urgently diagnostic methodology innovations are needed.

**Fig. 2:**
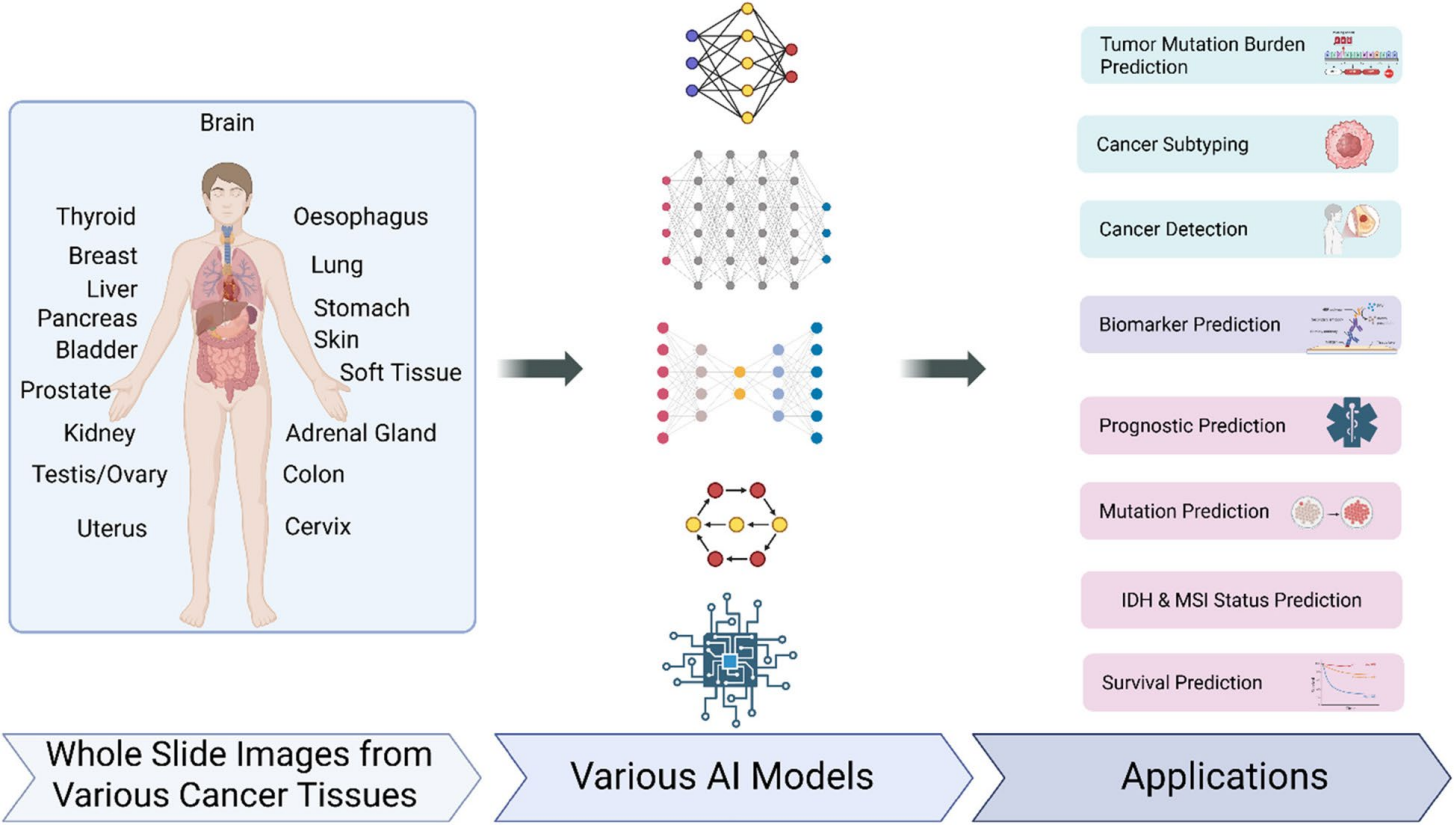
Whole slide images from different cancer tissues are processed using diverse AI models to enable key applications like cancer detection, subtyping, mutation and biomarker prediction, prognostic evaluation, and survival forecasting, advancing precision oncology through deep learning insights

Several AI models are being used for cancer detection imaging. These models include Prov-GigaPath [64], Owkin's models [65], CHIEF [66], and Google Deepmind AI [67]. Conventional AI models are trained to do particular tasks, such detecting cancer cells or forecasting treatment results. Nevertheless, these models require extensive training datasets, and their outcomes frequently fluctuate depending on the tissue type or imaging technique (Fig. 2). Usually, they are modified from computer vision models that were first created to recognize large objects. Self-supervised learning is a more-adaptable technique that trains AI models using unlabeled data and was shown to perform better on a variety of tasks. However, despite recent developments in self-supervised learning models, the widespread application of AI models for cancer diagnosis is still hampered by their limited generalizability and narrow task emphasis.

Y. Ma et al. (2025) [68] introduced HistoPathExplorer, a web-based tool for evaluating AI in histopathology. It standardizes datasets and metrics, enabling users to explore model performance and clinical relevance. A highlighted study used MIL on 1,065 CRC WSIs from the MCO dataset to predict microsatellite instability (MSI), a key biomarker, by aggregating features from artifact-free tiles via pretrained models [64]. With an AUROC of 0.91, the MIL-based method demonstrated high predictive accuracy, underscoring its potential as a non-invasive alternative to traditional biomarker testing in CRC. However, the study noted limitations, such as dependence on WSI quality and high computational demands, and suggested directions for future research to address these challenges.

Digital pathology has significantly advanced with the Prov-GigaPath concept in the context of cancer medical imaging. Prov-GigaPath is a foundation model created jointly by Providence Health System, Microsoft, and the University of Washington. Its purpose is to analyze gigapixel whole-slide pathology images in order to improve cancer diagnoses and patient care [69]. To capture both local and global context, the model employs a two-tier architecture: a tile encoder processes 256 × 256 pixel tiles from WSIs to extract local features, while a slide encoder aggregates these embeddings into comprehensive slide-level representations. This design enhances accuracy and efficiency by addressing the computational challenges posed by large WSIs. Leveraging Prov-Path, a large-scale dataset from the Providence Health Network with slides from over 30,000 patients and 31 major tissue types, Prov-GigaPath achieved top-tier performance across 26 pathology tasks. The dataset is more than twice the size of TCGA in patient count and over five times larger in tile volume, providing a robust foundation for model training [70].

Prov-GigaPath outperformed current models like the Hierarchical Image Pyramid Transformer (HIPT) in comparative studies, achieving state-of-the-art performance across 26 digital pathology tasks, including mutation predictions and cancer subtyping. Prov-GigaPath, for example, outperformed HIPT in mutation prediction tasks, as evidenced by its superior AUROC and area under the precision-recall curve (AURPC) scores. There are various advantages to incorporating Prov-GigaPath into medical imaging processes [71]. Because of its capacity to interpret gigapixel WSIs, tissue samples can be thoroughly analyzed, leading to better cancer diagnoses and more-individualized treatment plans. Precision immunotherapy relies on the model's ability to comprehend the tumor microenvironment (TME) by identifying both local and global patterns in pathology slides. Additionally, as an open-weight model, Prov-GigaPath promotes openness and cooperation throughout the scientific community, leading to improvements in digital pathology. But there are restrictions to take into account. The quality and diversity of the training data determine how well the model performs; biases or inconsistencies in the Prov-Path dataset may limit how broadly the model can be applied. Furthermore, processing gigapixel photographs requires a significant amount of CPU power, which could be problematic for organizations with weak infrastructure [70].

X. Wang et al. (2024) [66] designed a general-purpose ML framework called Clinical Histopathology Imaging Evaluation Foundation, or CHIEF, that can extract various features from pathology images for cancer diagnoses and evaluation. This was done in order to address the limited generalizability of some AI models in analyzing images from different populations and digitization methods. Using self-supervised learning and attention-based integration, it was trained on 60,000 WSIs from 14 cohorts and outperformed existing models in 11 cancers. CHIEF combines patch-level feature extraction with global representation learning, leveraging CTransPath and CLIP encoders. Validated on data from 24 hospitals, it demonstrated strong performance in prognosis, tumor origin detection, and cancer cell classification.

CHIEF outperformed ABMIL, CLAM, and DSMIL across 15 datasets and 11 cancers, with an AUROC of 0.9397—10–14% higher. Its pixel-level predictions closely matched pathologist evaluations and identified key mutations like TP53 and BAP1. Similarly, DeepMind’s CNN-based system enhanced breast cancer screening, reducing false positives by 5.7% and false negatives by 9.4% using large mammogram datasets. Together, CHIEF and Prov-GigaPath mark a shift from task-specific tools to scalable models excelling in prediction accuracy, subtyping, and biomarker detection—demonstrating the growing maturity of AI in cancer diagnostics [72]. The Lymph Node Assistant (LYNA), another product of Google's AI research, analyzes histopathological slides to identify metastatic breast cancer. With a 99% accuracy rate in diagnosing metastatic cancer, LYNA outperformed human pathologists, particularly in spotting tiny metastases, which are sometimes difficult to find [73].

Alternative AI models such as AI Initiatives at the University of Pittsburgh assist pathologists in diagnosing prostate cancer, and the University of Pittsburgh Medical Center (UPMC) has used AI technologies such as Galen Prostate™ from Ibex Medical Analytics. In order to detect cancer and evaluate characteristics like Gleason grades, perineural invasion, and tumor sizing. Galen Prostate uses DL algorithms that have been trained on large datasets, including rare prostatic cancers. Northwell Health created iNav, an AI-powered diagnostic tool, to improve pancreatic cancer early diagnosis and treatment [74]. iNav detects patients with radiographic signs of pancreatic cancer through radiology data analysis, enabling timely care. It uses an NLP classifier trained to recognize phrases in radiology reports linked to pancreatic cancer, scanning for language patterns and keywords tied to masses or lesions. When indicators appear, iNav flags them for further medical review. Given pancreatic cancer’s late detection and poor prognosis, iNav improves early detection by proactively analyzing imaging. It cut the diagnosis-to-treatment time by 50%, tripled biospecimen study participation, and increased referrals to multidisciplinary clinics, improving care and research opportunities. An improved DL model called Dual-Domain Residual-based Optimization NEtwork (DRONE) [75] was developed. DRONE reduces artifacts and boosts image quality by integrating image and data domains (sinogram). It has three modules: the embedding module expands sparse sinogram data via an encoder-decoder network, enriching inputs; the refinement module improves initial images using a deep CNN; and the awareness module ensures consistency between sinogram and reconstructed images through regularization, integrating outputs from the other two modules. DRONE addresses sparse-view CT challenges by combining outputs across modules. Its performance evaluated using PSNR, SSIM, and RMSE surpassed conventional and other DL methods in reconstruction accuracy, feature retention, and edge clarity. The integration of AI and ML into cancer diagnostics has markedly improved accuracy, speed, and treatment personalization. AI excels at analyzing complex datasets, leading to more accurate diagnoses and faster treatment initiation, which improves outcomes. It also supports personalized medicine by integrating genetic and clinical data to tailor treatments (Table 2). Developing AI/ML models for cancer detection involves key steps. Data collection requires diverse, high-quality datasets, including imaging, genomics, and patient histories. Preprocessing ensures data consistency via normalization, augmentation, and annotation. Model selection is task-specific CNNs for images, RNNs or LSTMs for sequential data, and decision trees for classification. Models are trained on large datasets and validated regularly to enhance accuracy. CNNs effectively analyze images like MRIs and mammograms; LSTMs and RNNs process sequential clinical data; decision trees and RFs support diagnostic decision-making. These models have demonstrated strong performance in cancer detection.

**Table 2:** Imaging-based artificial intelligence (AI) diagnostics in cancer

AI Diagnostic Tool	Cancer Type	Imaging Modality	AI Model Used	Dataset Used	Performance Benchmark	Clinical Validation Status	Limitations/Challenges	References
CHIEF	Esophagus, stomach, colon, prostate	Histopathology (WSIs)	Transformer-based	Millions of WSIs	Accuracy up to 96%	Clinical trials ongoing	Computational demand, dataset bias	[66]
Google DeepMind AI	Breast cancer	Mammography	CNN	25,000 + scans (UK & US)	Exceeded expert accuracy	Validated clinically	Requires large labeled datasets	[67]
University of Pittsburgh AI	Prostate cancer	Histopathology (WSIs)	CNN	TCGA, hospital data	Sensitivity 98%, specificity 97%	Clinical trials ongoing	Generalizability challenges	[76]
Radiomics Approach	Lung, head-neck cancers	CT imaging	Radiomics + SVM, RF	1019 patient scans	Improved prognostics	Retrospective validation	Interpretability	[55]
AI Breast Cancer Screening	Breast cancer	Mammography	CNN	100,000 + mammograms	17.6% higher detection rate	In clinical use (Sweden)	False positives/negatives	[77]
iNav	Pancreatic cancer	MRI, CT	Deep learning, reinforcement Learning	Northwell dataset	Diagnosis-treatment time halved	FDA review ongoing	Computational demands	[78]
AI Retroperitoneal Sarcoma Grading	Retroperitoneal sarcoma	CT imaging	CNN + Radiomics	Multi-center validation	82% accuracy	Clinical validation ongoing	Needs external validation	[79]
Owkin's Models	Mesothelioma, various	Histopathology (WSIs)	CNN	Diverse datasets	Improved outcomes prediction	Clinical validation ongoing	Clinical integration issues	[80]
DRONE: Dual-Domain Residual-based Optimization NEtwork	Various	CT, MRI, PET, SPECT, ultrasound	Deep neural networks	Diverse datasets	Enhanced image quality	Research phase	Computational complexity	[75]
Richard J. Cote AI	Various	Histopathology	Deep learning	Diverse datasets	Better metastasis predictions	Research ongoing	Data privacy, dataset needs	[81]
Deciphex's Diagnexia & Patholytix	Various	Histopathology	AI platforms	Health system collaborations	Improved diagnostic efficiency	Clinical implementation	Data integration, regulations	[82]
PSMA PET/CT Scan	Prostate cancer	PET/CT	Advanced imaging techniques	102-patient trial	Precise cancer spread detection	Phase 2 clinical trial	Comparative analysis needed	[83]
TD-CNN-LSTM-LungNet	Lung diseases	Ultrasound videos	Explainable AI	Lung ultrasound dataset	96.57% accuracy	Research phase	Generalization, modality expansion	[84]
AI MRI Brain Tumors	Gliomas, glioblastomas	MRI	CNN, U-Net	BraTS datasets	Improved segmentation	Clinically implemented partially	Scanner variability	[85, 86]
AI PET/CT Lung Cancer Staging	Lung cancer	PET/CT	Deep learning, radiomics	NLST dataset	Higher staging accuracy	Research ongoing	Imaging quality, cost	[87]
AI Colorectal Cancer Diagnosis	Colorectal cancer	Endoscopy (NBI, WLI)	YOLO, ResNet CNN	Endoscopic datasets	Sensitivity 96.3%, specificity 93.1%	Multi-system validation	Procedure variability	[88, 89]
AI Thyroid Nodule Classification	Thyroid cancer	Ultrasound	DenseNet, ResNet	Hospital datasets	Improved accuracy vs radiologists	Clinical validation ongoing	Device standardization	[90]
DNPR model	Pancreatic cancer	CT imaging	CNN, radiomics	Multi-center CT dataset	≈90% early-stage accuracy	Validation studies ongoing	Small dataset size	[91]

*WSIs* Whole-slide images, *CNN* Convolutional neural network, *TCGA* The Cancer Genome Atlas, *CT* Computed tomography, *SUM* Score-Unifying Metric, *RF* Random forest, *MRI* Magnetic resonance imaging, *FDA* Food and Drug Administration, *PET* Positron emission tomography, *SPECT* Single-photon emission CT, *NCST* Non-Contrast CT, *NBI* Narrow-band imaging, *YOLO* You Only Look Once

### AI in genomics and biomarker discovery

**Fig. 3:**
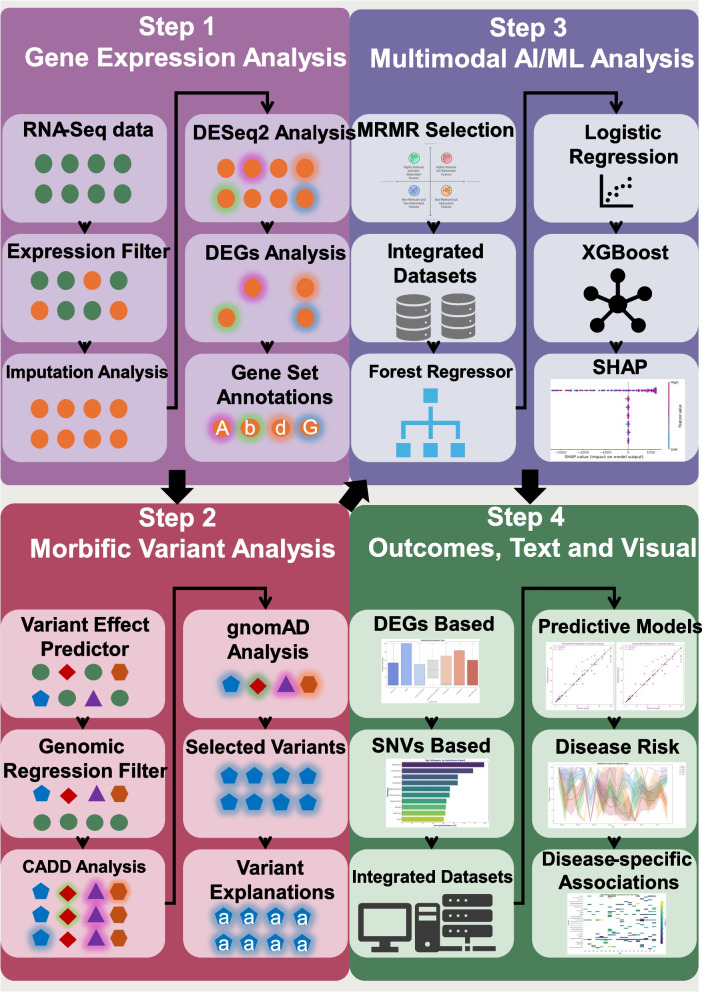
Integrative pipeline combining gene expression, variant analysis, and AI/ML modeling. It starts with RNA-Seq-based differential gene analysis, followed by morbid variant filtering, multimodal machine learning, and finally outputs predictive models, risk estimations, and disease-specific visual associations for precision medicine

Advancements in proteomics, genomics, and combinatorial chemistry have led to numerous chemical and biological databases, greatly enhancing our understanding of cancer molecular biology. Clinically, this knowledge can transform cancer assessment and treatment. However, identifying therapeutically relevant insights from vast raw genetic data remains a challenge. Researchers have applied AI to identify cancer subgroups based on genes, mRNA, and miRNA clusters (Fig. 3). Using deep flexible neural forest models and stacked autoencoders (AEs), mRNA, miRNA, and DNA methylation data were integrated to classify ovarian, breast cancers, and glioblastomas into subtypes [92, 93].

Both supervised and unsupervised learning were applied to RNA, miRNA, and methylation data in hepatocellular carcinoma (HCC), revealing survival-associated consensus driver genes and two distinct patient subgroups. Multi-omics integration of proteomics and metabolomics data also stratified breast cancer patients into low- and high-risk groups. For subtyping, AEs and multiple-kernel frameworks were used [94]. AI plays a critical role in stratifying patients into prognostic and survival-based subgroups, enabling early cancer detection and progression forecasting. In neuroblastoma, gene expression and copy number alteration data helped classify subgroups [95]. For colorectal cancer (CRC) relapse prediction, integrated features included copy number variations, metabolomics, miRNA, and gene expression data [96]. he MRMR technique identified survival-related features in ovarian cancer [97]. Surviving breast cancer was also predicted using neural networks trained with DL [98]. The SALMON method combined multi-omics data and conventional biomarkers via eigengene matrices of co-expression networks to identify key genes and cytobands [99]. Additionally, a kernel-based ML approach was used to assess the predictive value of transcriptomic, epigenomic, and genomic data for different tumors [100]. When clinical criteria are taken into account, this method has demonstrated notable gains, although its effectiveness varies depending on the type of cancer.

Technological advances have made it possible for software developers and health researchers to closely work together to use multifactor analyses to enhance predictions. These assessments are reported to be much more accurate than the actual numbers. Creating models that use AI algorithms for cancer detection and prognosis is becoming a higher priority for researchers. These tactics are currently being used to improve the accuracy of cancer prognoses that are diversified and recurrent, and promote survival [101].

In order to learn more about the molecular basis of the disease, clinical oncology research has primarily focused on fully understanding the mechanisms driving the proliferation of cancer cells. Additionally, its goal is to use computational biology to manage enormous volumes of data from millions of relevant cases in order to combat rising worldwide mortality rates linked to cancer. Furthermore, it is anticipated that the use of AI for clinical decision-making will improve the use of NGS and high-resolution imaging for early illness identification and prediction [100]. AI has the potential to greatly increase the precision and promptness of disease detection and prognosis by utilizing these cutting-edge technologies [102]. AI has the potential to produce new biomarkers for cancer diagnosis. Building a system that is sufficiently trained to correctly assess whether a patient will need immunotherapy is the aim of AI functionality. AI can estimate which immunotherapeutic medications will have the largest impact on a patient's recovery and identify patients who need additional testing, such as whole genome spectroscopy. With the help of validated real-world case studies, AI is increasingly being used in the medical field with the goal of successfully overcoming obstacles of correctly detecting various cancer types. A comprehensive description of the framework needed for AI to operate as planned is also included [103]. AI-driven biomarker discovery uses sophisticated computer methods to examine large, intricate biological datasets. SVMs and RFs are two popular supervised learning techniques that are used to precisely stratify patients by classifying them according to biomarker profiles [104]. These models can recognize important biological characteristics linked to therapy responses or illness progression since they are trained on labeled datasets. Unsupervised learning approaches, on the other hand, such as clustering algorithms (like k-means and hierarchical clustering), reveal hidden patterns in genomic data and facilitate finding new biomarker groupings without predetermined labels [105]. Numerous AI models are used in genomics and biomarker discovery to process large-scale genomic datasets, find disease-associated biomarkers, and support personalized medicine (Table 3). These models include DeepVariant [106] (Google), AlphaFold [107], IBM Watson for Oncology (WFO) [108], AI-Driven Liquid Biopsy Analysis, CancerSEEK AI [109], PRS-AI [110], and AI for Drug Response Prediction [111].

**Table 3:** AI in genomics and biomarker discovery

AI Tool	Application	AI Model Used	Dataset Used	Performance Benchmark	Clinical Validation Status	Limitations/Challenges	Data Type Processed	Regulatory Status	Use in Clinical Practice	Reference
DeepVariant (Google)	NGS variant calling	Deep learning (CNN)	1000 Genomes, Genome in a Bottle	Higher accuracy than traditional methods	Used in research & clinical settings	Requires extensive computational power	Genomic sequencing	Research use only	Limited use in hospitals	[106]
AlphaFold	Protein structure prediction (proteomics)	Transformer-based deep learning	Protein Data Bank (PDB)	RMSD accuracy improvement	Validated on CASP challenges	Limited to known protein sequences	Proteomics	Research use only	Used in pharmaceutical R&D	[107]
IBM Watson for Oncology	Precision oncology	NLP, ML-based decision support	Large genomic & clinical datasets	Improved treatment recommendations	Used in select hospitals	Limited explainability, bias concerns	Clinical & genomic data	Some FDA-approved components	Used in cancer treatment centers	[112]
AI-Driven Liquid Biopsy Analysis	ctDNA & CTC-based cancer detection	ML, CNN-based	Large liquid biopsy datasets	Higher specificity & sensitivity for early detection	Research phase, some trials ongoing	Standardization issues in clinical application	Liquid biopsy, ctDNA	Not FDA-approved yet	Limited trials for early detection	[113]
CancerSEEK AI	Multi-cancer blood test	ML-based ensemble models	CancerSEEK cohort (10,000 + patients)	Early detection of 8 + cancers	Clinical trials ongoing	Cost of implementation, false positives	Circulating biomarkers	Not FDA-approved yet	Pilot trials in screening programs	[109]
PRS-AI	Personalized risk assessment	AI-based polygenic risk scores (PRSs)	UK Biobank, large GWASs	More-accurate risk stratification	Used in some precision medicine initiatives	Ethical concerns, population bias	Genomic risk profiling	Not FDA-approved yet	Early-stage implementation in genetic counseling	[114]
AI for Drug Response Prediction	Precision oncology drug matching	ML, deep reinforcement learning	GDSC, CCLE datasets	Improved patient-specific therapy recommendations	Limited clinical trials	Requires extensive validation	Genomic & drug response data	Not FDA-approved yet	Limited use in precision oncology	[115, 116]

*NGS* next generation sequencing, *CNN* convolutional neural network, *RMSD* root mean squared deviation, *CASP* critical assessment of protein structure prediction, *R&D* research and development, *NLP* natural language processing, *ML* machine earning, *FDA* Food and Drug Administration, *ctDNA* circulating tumor DNA, *CTC* circulating tumor cell, *GWASs* genome-wide association studies, *GDSC* Genomics in Drug Sensitivity in Cancer, *CCLE* Cancer Cell Line Encyclopedia

Google’s DeepVariant, a deep CNN for detecting genetic variants in NGS data, transforms sequencing reads into pileup images and classifies them using the Inception architecture for accurate genotype predictions. Evaluated on Genome in a Bottle benchmarks, its performance was assessed using precision, recall, and F1-score across diverse genomic contexts. DeepVariant outperformed traditional methods, especially in complex regions.

DeepMind’s AlphaFold predicts protein structures from amino acid sequences using deep learning, evolutionary data, and attention mechanisms. Its performance in CASP contests showed near-experimental accuracy, advancing structural biology and drug discovery. H. Sun et al. (2025) [117] showed AlphaFold 3’s role in identifying cancer biomarkers in uveal melanomas via cytokine pathway analysis, combining AlphaFold predictions with scRNA-Seq, docking, and enrichment studies. It effectively revealed treatment-relevant biomarkers. Somashekhar et al. (2017) [118, 119] assessed IBM Watson for Oncology (WFO) on 638 breast cancer cases, comparing its treatment suggestions with Manipal Multidisciplinary Tumor Board (MMDT). WFO categorized recommendations as REC, FC, or NREC, using ML and NLP to mine medical data. Overall concordance was 73%, with 80% in non-metastatic and 45% in metastatic cases. Triple-negative agreement was 67.9%; HER2-negative, 35%. WFO generated recommendations in 40 s, compared to 12–20 min manually, aiding biomarker-based decision-making despite limitations across cancer subtypes.

Jin et al. (2025) [120] explored computational techniques for early pancreatic cancer (PC) detection. ML models like RFs, SVMs, and DL were applied to complex datasets. NGS and GWAS helped identify key mutations (e.g., *TP53*, *KRAS*). AI-based CDSSs used Bayesian networks for personalized risk and treatment. Radiomics via CNNs and CEH-EUS imaging improved diagnosis, while liquid biopsies detected ctDNA, CTCs, miRNAs, and exosomes. Multi-omics integration enhanced precision medicine for early diagnosis and personalized care. Sud et al. (2021) [121] examined polygenic risk scores (PRSs) for cancer susceptibility. PRSs aggregate multiple variants to estimate risk but vary in accuracy by cancer type. For PC, PRSs achieved an AUC of ~ 0.67, but clinical utility was limited in rare cancers due to minimal absolute risk increases. Combining PRSs with non-genetic risk factors yielded only slight gains in predictive power. The effectiveness of PRSs for guiding interventions like screening or prevention remains uncertain and requires further study.

### AI-Powered liquid biopsies and early cancer detection

**Fig. 4:**
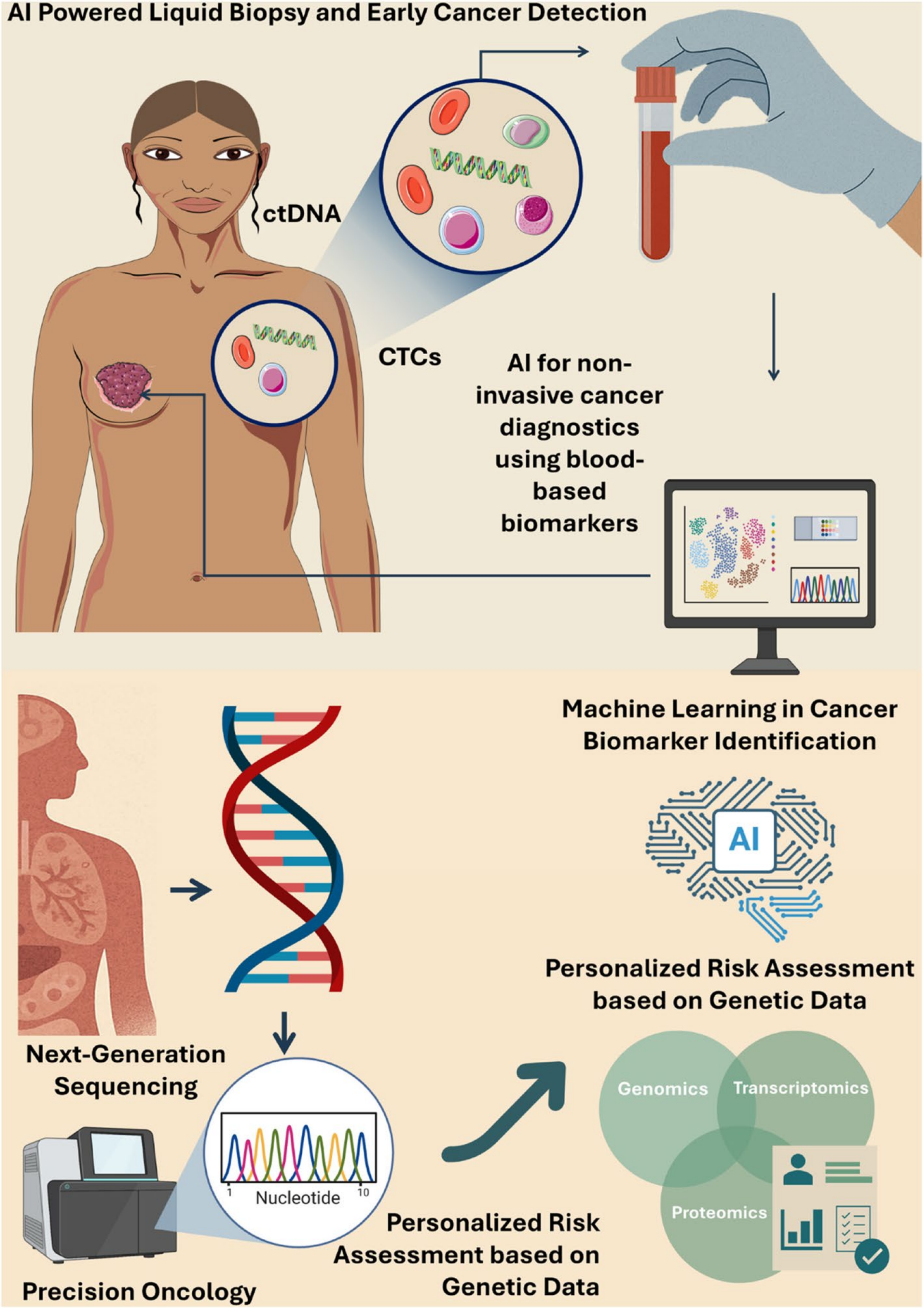
AI-powered liquid biopsy and genomic technologies for early cancer detection and personalized oncology. It highlights the use of circulating biomarkers (ctDNA, CTCs), next-generation sequencing, and AI/ML models to identify cancer biomarkers and assess individual risk using multi-omics data for precision treatment planning

The identification and examination of liquid biopsy biomarkers, such as circulating tumor cells (CTCs) and circulating tumor DNA (ct)DNA, have made tremendous strides in the last 10 years. Their clinical utility in early cancer detection, disease monitoring, and therapy response evaluations have earned them acclaim (Fig. 4). The advent of liquid biopsies is beneficial since it provides a quick, real-time monitoring method that is minimally invasive and may be an alternative to conventional tissue biopsies. In environments with limited resources, the optimal liquid biopsy platform should correctly reflect the molecular heterogeneity of the patient's illness in addition to extracting more CTCs or ctDNA from a small sample volume [122].

In liquid biopsies, small amounts of biofluids are collected to analyze components produced by cancer cells. Rich supplies of cancer biomarkers can be found in blood, saliva, urine, and cerebrospinal fluid. These biomarkers can exist in free form or be linked to other fluid-secreted structures. Liquid biopsies may make it easier to conduct dynamic studies of molecular or cellular biomarkers. Accurate early-stage diagnosis and prognosis, tracking the course of the disease, evaluating the effectiveness of certain treatments, and determining therapeutic goals for drug development are all made possible by liquid biopsies [123].

Additionally, liquid biopsies facilitate dynamic studies of cellular or molecular indicators. CTCs and ctDNA are two biofluid components that were shown in studies to be essential for early-stage cancer detection [124]. The primary tumor site releases components including CTCs and ctDNA into the bloodstream, which aids in the spread of cancer. However, they are difficult to detect and evaluate due to their great degradability and low concentration (1–1000 CTCs/mL) [125]. A major obstacle to any separation and characterization strategy in cancer research is the physical similarity of CTCs to certain white blood cells (WBCs), phenotypic heterogeneity, and the epithelial-to-mesenchymal transition (EMT). The EMT is a process through which cancer cells pass to help them separate from the main tumor and enter the bloodstream [126]. EMT entails the acquisition of mesenchymal traits, such as the expression of the cytoskeletal protein, vimentin, and the loss of epithelial traits, such as downregulation of the adhesion molecule, E-cadherin. Vimentin is linked to cancer cell invasiveness and macrophage-secreted interleukin (IL)-35 and was reported to be overexpressed in a variety of cancers, including breast cancer and extrahepatic cholangiocarcinomas. The transcription factors SNAI1 (also called SNAIL), SNAI2 (SLUG), TWIST1, and FOXC2 can regulate the EMT process, which is regulated by transforming growth factor (TGF)-β, Wnt, and Notch signaling [127, 128]. Single cancer cells or groups of tumor cells may be involved in this invasion process. While intrinsic tumor hypoxia may trigger the intravasation of cell clusters, other alternative routes likely also play a role. TGF-β signaling can mediate the intravasation of single cells [129].

The Circulating Cell-free Genome Atlas (CCGA) project is a premier example of a population-scale investigation that combines ML and cancer cell-free (cf)DNA [130]. It seeks to ascertain if ML can use genome-wide cfDNA sequencing data to detect and localize numerous cancer types with high specificity. Whole-genome bisulfite sequencing (WGBS) was reported to perform better than whole-genome sequencing (WGS) and targeted genome sequencing approaches in terms of genome-wide methylation patterns in the first CCGA sub-study [131]. Custom models identify methylation patterns per location as being comparable to those originating from a certain form of cancer in the second sub-study. Two logistic regression ensembles carry out tissue of origin localization and additional cancer/non-cancer sample classification.

Building upon these ideas, J. Li et al. (2021) [132]) created a unique method called DISMIR that uses low-depth cfDNA sequencing data to provide sensitive and reliable cancer detection. This method combines plasma cfDNA data from WGBS and WGS. The "switching region" idea, a novel feature engineering technique used in DISMIR, efficiently identifies cancer-specific differentially methylated regions that support individual read source predictions. The site of malignancy and tumor load can be predicted by mapping cfDNA reads back to their source. To determine the origin of each read and the malignancy status, DISMIR uses a DL model that incorporates DNA sequences and methylation states. This model does a good job of detecting hepatocellular cancer. Early cancer detection can be aided by cfDNA data and ML techniques. In order to help distinguish non-cancer controls and patients with early-stage CRC, Wan et al. (2019) [133] created computational methods that can identify correlations between cfDNA profiles and the cancer status. By counting the number of fragments that overlapped each known protein-coding gene, they converted WGS data from cfDNA into pertinent input features. They then normalized the data to take feature-length, read depth, and sequence-content biases into account.

In order to improve the efficiency of models in making numerous clinically relevant decisions, future research in cfDNA analysis could also look into alternatives to the use of ML algorithms as models, such as ensemble and hybrid models, various neural network structures (such as CNNs, AEs, and RNNs), and training methods like transfer learning.

Mgbole (2025) [134]examined how CTCs, cfDNA, miRNAs, and protein biomarkers can be used to detect metastatic cancer using DL models, namely CNNs [135] and RNNs [136]. To guarantee the generalizability and robustness of the model, the study incorporated multimodal data from extensive, multicenter datasets that included blood samples from various patient cohorts. The methodology included CNN-based image recognition for immunofluorescent CTC detection [137], signal processing techniques for preprocessing biomarker data, AEs and deep neural networks (DNNs) for feature extraction, and RNNs for analyzing temporal variations in cfDNA mutations for early metastasis predictions. In order to increase the predictive accuracy by utilizing spatial and sequential biomarker data, the study used an ensemble model technique that combined CNNs and transformer-based DL architectures and DL models to maximize classification performance. The use of transfer learning, which reduced data dependency and computational costs by fine-tuning pretrained models on sizable biology datasets for predicting metastatic cancer, was a crucial component of the work. The study outperformed conventional biomarker-based diagnostics by demonstrating high sensitivity (> 90%) and specificity (> 95%) in detecting metastatic signals from blood samples [138]. According to the results, AI-driven liquid biopsy analyses can lessen the need for invasive tissue biopsies, enable individualized treatment plans, and greatly increase early detection rates. In order to ensure that neural network decisions are in line with biological and clinical expectations [113], the study emphasized the use of explainable AI (XAI) tools like Grad-CAM [139] and SHAP (Shapley Additive Explanations) [140] for model transparency. To improve diagnostic precision and individualized cancer treatment, future prospects involve merging multi-omics datasets, which combine liquid biopsy AI models with genomics, transcriptomics, and proteomics (Table 4).

**Table 4:** Artificial intelligence (AI)-powered liquid biopsy and early cancer detection

AI Diagnostic Tool	Cancer Type	77	AI Model Used	Dataset Used	Performance Benchmark	Clinical Validation Status	Limitations/Challenges	Reference
AI for ctDNA & CTC Analysis	Metastatic breast cancer	Circulating tumor (ct)DNA, circulating tumor cells (CTCs)	Deep learning (CNN, RNN)	Large-scale multicenter datasets	High sensitivity/specificity in detecting ctDNA	A proof of principle study	Requires large cohort validation; costly sequencing	[141]
Galleri by GRAIL	Multiple cancers	Methylation signatures in cfDNA	Deep learning + AI-driven classifiers	50,000 + participant datasets	Identifies over 50 cancer types with high specificity	Implemented in screening programs	False positives in non-cancer cases	[142]
Multi-task generative AI model, (Orion)	Early stage lung cancer	Circulating orphan non-coding RNAs	Semi-supervised variational auto-encoder	Retrospective and prospective studies	Overall sensitivity of 94% (95% CI: 87%–98%) at 87% (95% CI: 81%–93%) %	Ongoing research	Regulatory and reimbursement challenges	[143]
AI for AI-Integrated Liquid Biopsy	Prostate, breast, lung cancers	miRNA, proteomics, ctDNA	Multi-modal deep learning	Large-scale cancer registries	Improved multi-cancer detection	Pending clinical deployment	Data heterogeneity across populations	[144]
Array-based genome-wide DNA methylation profiles	Central nervous system Tumors	DNA methylation	Deep learning	Population-based cancer screening datasets	High predictive accuracy across early-stage cancers	Screening program integration underway	Cost-effectiveness concerns	[145]
Acoustic Separation and Concentration of Exosomes and Nucleotide Detection: ASCENDx	Colorectal cancer	Circulating exosomal microRNA biomarkers	AI-based feature extraction	Multicenter exosome studies	Identifies tumor-specific exosomes with 95.8% sensitivity and 100% specificity	Preclinical validation	Standardization of exosome isolation	[146]

*CNN* Convolutional neural network, *RNN* Recurrent neural network, *miRNA* microRNA, *CI* Confidence interval

Klein et al. (2021) [147] evaluated GRAIL’s Galleri test, an AI/ML-powered multi-cancer early detection (MCED) tool analyzing cfDNA methylation patterns. Part of the CCGA study (NCT02889978), this large clinical validation sub-study included 4,077 participants—2,823 cancer patients and 1,254 confirmed non-cancer controls (1-year follow-up). The test demonstrated high specificity (99.5%) and stage-dependent sensitivity: 16.8% (stage I), 40.4% (II), 77.0% (III), and 90.1% (IV), with an overall sensitivity of 51.5%. For 12 high-mortality cancers, stage I–III sensitivity reached 67.6%. In 88.7% of true positives, the test accurately identified the cancer signal origin, detecting over 50 cancer types. These results support Galleri’s potential as a blood-based complement to current single-cancer screening methods, enhancing early detection across diverse cancers [148].

Karimzadeh et al. (2024) [143] developed Orion, a multi-task generative AI model for analyzing circulating orphan non-coding RNAs (oncRNAs) to enhance liquid biopsy-based early detection of non-small cell lung cancer (NSCLC). The study used serum from 1,050 treatment-naive individuals—419 NSCLC patients (stages I–IV) and 631 age-, sex-, and BMI-matched controls. RNA was extracted from 0.5 mL serum samples and sequenced (avg. depth: 19.8 M 50-bp reads).

Orion employs a semi-supervised variational autoencoder (VAE) with two arms: one modeling oncRNA expression and another processing annotated short RNAs. Integrating classification and contrastive learning, Orion adjusts for library variability and improves label prediction. It achieved an AUROC of 0.97 (95% CI: 0.96–0.98) and 94% sensitivity (CI: 91%–96%) at 90% specificity in tenfold cross-validation. Sensitivity for stage I and T1a–b tumors was 90% and 87%, respectively. On an independent 20% validation set, Orion retained strong performance (AUROC: 0.95; sensitivity: 92% at 90% specificity), outperforming SVM, ElasticNet [149], and XGBoost [150] by about 30% in the separate validation dataset.

The use of multi-modal DL approaches that incorporate information from several sources, such as imaging investigations, clinical records, and genomic sequences, was highlighted in a review by D. Huang et al. (2024) [151]. Using RNNs and CNNs, multimodal DL models integrate imaging, clinical, and liquid biopsy data to improve early lung cancer detection and prognosis (Table 4). Though lacking dataset specifics, the review highlighted AI’s potential in multi-omics-driven personalized care and its role in advancing precision oncology. Viet et al. (2024) [152] examined the relationship between heavy tobacco use (≥ 10 pack years) and oral squamous cell carcinoma (OSCC) and its potential for early cancer diagnosis by integrating multi-omics data with DL techniques. In order to differentiate heavy smokers from non-smokers, researchers used The Cancer Genome Atlas (TCGA) cohort (*n* = 257) and an internal cohort (*n* = 40) to identify 13 differently expressed genes (*IGHA2, SCG5, RPL3L, NTRK1, CD96, BMP6, TFPI2, EFEMP2, RYR3, DMTN, GPD2, BAALC, and FMO3*) and three differentially methylated genes (*GPR15*, *GNG12*, and *GDNF*). Significant disruptions in pathways linked to platelet activation, cell adhesion, and extracellular matrix architecture were found by functional pathway studies, linking these molecular changes to the pathophysiology of OSCC in smokers. The Slideflow [153] pipeline was used to handle 203 TCGA full-slide pictures that were stained with H&E for histological evaluation. After being labeled by skilled pathologists, the ROIs were separated into tiles with 299 × 299 pixels and then stained, normalized, and enhanced. Using the Xception [154] architecture and pretrained weights from ImageNet, a DL model was trained to predict the smoking status and 5-year mortality. During three-fold cross-validation, patient-level AUROCs for smoking status predictions ranged 0.49–0.62, while for mortality predictions, they ranged 0.48–0.54. By combining clinical characteristics, discovered genetic markers, and histological modeling, the combined method was able to predict OSCC patients' 5-year mortality with a c-statistic of 0.9409. The potential of AI-driven methods to improve cancer diagnoses and prognoses was further highlighted by the use of DL for histology data [155].

Exosomes are tiny extracellular vesicles released by cells. Because they include proteins, lipids, and nucleic acids that are representative of the cell in which they originate, they have become essential biomarkers in liquid biopsies for early cancer diagnoses. In order to improve the sensitivity and specificity of exosome-based cancer diagnoses, recent developments have combined biosensor technologies with AI, specifically DL models like CNNs [156]. The aptasensor employs a multi-probe recognition approach, using methylene blue (MB)- and ferrocene (Fc)-functionalized aptamers as signal units and CD63, HER2, and EpCAM aptamers as capture units. This arrangement improved exosome analysis for screening and prognosis by distinguishing breast cancer subtypes. AI-enhanced biosensors use DL algorithms to assess volatile organic compounds (VOCs) in breath samples, allowing for non-invasive diagnostics. One example of this is the electrochemical gas sensor with a graphene-Prussian blue layer designed for lung cancer (LC) detection. Furthermore, by processing sensor data using a strong neural network trained on PC biomarker signatures, the MOOSY-32 electronic nose (EN) system with AI improved non-invasive prostate cancer diagnoses [157]. The scarcity of structured datasets in biosensing is a serious obstacle to the broad use of DL methods, which necessitate sizable datasets. AI-assisted biosensors were combined with surface-enhanced infrared (IR) absorption (SEIRA) spectroscopy to help identify biomarkers by dynamically monitoring protein interactions with biomolecules including lipids and nucleic acids [158]. AI integration with spectroscopic methods (NMR, MS, IR) enhances biomarker detection across diseases. DL-powered biosensors and electronic olfaction/gustation automate biochemical data analysis, enabling accurate, expert-independent, point-of-care cancer diagnostics.

#### AI in cancer treatment and therapy optimization

The prospect of creating new anticancer treatments or at least directing their development to reduce failure rates and approval times, is one of the most exciting possible uses of AI in cancer. There are unmistakable indications that some neural network-type autoencoders, for instance, can learn to represent a group of molecules with particular activities and generate new structures with related activities. Additionally, AI can be utilized to precisely predict the mechanism of action of anticancer drugs, enhancing the likelihood of clinical success and enabling precise preclinical and clinical positioning (Table 5). Similarly, as the number of anticancer medications keeps increasing, predicting successful drug combinations has grown into a challenging combinatorial problem that AI may be able to resolve [159].

**Table 5:** Artificial intelligence (AI) for treatment planning and decision support in oncology

AI System	Cancer Type	Application Area	AI Model Used	Dataset Used	Performance Benchmark	Clinical Validation Status	Limitations/Challenges	Reference
IBM Watson for Oncology	Multiple	AI-based CDSS for oncologists	NLP + ML	Large clinical datasets	Recommends treatments similar to oncologists in > 90% of cases	Used in hospitals globally	Data bias, interpretability issues	[160]
Tempus AI	Multiple	Precision oncology treatment recommendations	Deep learning	Genomic + clinical data	Improved therapy matching accuracy	Clinical implementation in progress	Data integration challenges	[161]
Flatiron Health AI	Multiple	AI-driven clinical decision support	ML & real-world data (RWD) models	EHR data from 2 M + patients	Enhanced oncologists’ decision-making	Widely used in oncology centers	Data privacy, standardization issues	[162]
DeepMind's AlphaFold in Oncology	Multiple	Drug discovery + precision medicine	Transformer-based AI	Protein structure databases	Improved drug-target predictions	Research phase	Needs clinical validation	[163]
Paige AI	Prostate & other cancers	AI-assisted pathology for treatment guidance	CNN + transformer	Histopathology WSI datasets	> 95% accuracy in tumor detection	FDA-approved for diagnostics	Requires integration with CDSS	[164]
RaySearch RayStation	Multiple	AI-driven radiotherapy planning	Deep learning	Radiotherapy treatment datasets	Enhanced dose predictions & adaptive radiotherapy	Used clinically in radiation oncology	Computational cost, regulatory compliance	[165]
Arterys Oncology AI	Lung, liver, brain cancers	AI-based clinical decision support	Deep learning	MRI, CT datasets	Faster and more-precise tumor detection	FDA-cleared	Image standardization issues	[165]
Varian Ethos AI	Multiple	Personalized adaptive radiotherapy	AI-driven adaptive radiotherapy model	Real-time patient imaging	Reduced treatment times, optimized doses	In clinical use	High cost, implementation complexity	[166]
OncoKB	Multiple	Precision oncology knowledge base	ML-based variant classification	Genomic sequencing data	Assists in identifying actionable mutations	Integrated into clinical workflows	Requires constant updates	[167]
Qure.ai Oncology AI	Lung, brain, breast cancers	AI for radiology-based treatment planning	Deep learning (CNN)	Multi-center radiology datasets	Outperformed radiologists in lesion detection	CE-certified, clinical trials ongoing	Black-box AI issues	[168]

*CDSS* Clinical decision support system, *HER* Electronic health record, *NCP* Nursing care plan, *ML* Machine learning, *CNN* Convolutional neural network, *WSI* Whole-slice image, *FDA* Food and Drug Administration, *MRI* Magnetic resonance imaging, *CE* European Community

### AI in treatment planning and decision support

The use of AI to resolve medical problems has long been hailed as a disruptive and near-future development. It has a lengthy history that began in the 1970s when clinical decision support systems (CDSSs) needed human input to choose qualities for these expert systems and supply rules for decision-tree approaches [169]. CDSSs based on AI emerged with the technical assistance of big data and ML. CDSSs assess drug efficacy, product accessibility, adverse reactions, patient financial status, and medical insurance types by combining various medical records, literature, and clinical research data. They then offer tailored recommendations to assist clinicians in optimizing treatment plans. AI's uses have grown beyond everyday problem solving to include medical professional domains like pathology diagnosis, image diagnosis, clinical treatment decision-making, prognosis analysis, and new drug screening (Table 5).

CDSSs based on AI technology have not fully achieved human–computer interactions in clinical practice as image-aided diagnosis systems because the ethics of applying AI as an emerging technology in clinical decision-making have not been thoroughly established. The Chinese Society of Clinical Oncology-Artificial Intelligence (CSCO AI), Watson for Oncology (WFO), and other organizations are now using and promoting CDSSs globally [170].

As the first commonly used CDSS in the field of cancer, WO [171] progressively gained global recognition in the areas of gynecological, lung, colon, rectal, breast, and stomach cancers. Medical personnel just need to enter a case's structured data according to the WFO system. The technology will produce extremely consistent evidence and the most conventional treatment strategy for the particular situation in less than a minute [108].

AI-based CDSSs simulate human reasoning to support clinical decisions, using ML models like DL, SVMs, LR, and ANNs. Built on structured medical data, they reduce errors, response times, and reliance on memory, enhancing safety, quality, and treatment efficacy.

Different from WFO, the CSCO AI system was established under the CSCO platform using the CSCO database and guidelines. The CSCO AI system mainly builds different knowledge maps based on schemes in CSCO guidelines [172]. When doctors search for relevant information, it locates the knowledge map and outputs results according to key information. Similarly, it is also updated in real time with guidelines to ensure the timeliness of the system.

Tempus is transforming precision oncology through AI and ML-powered individualized therapy recommendations. By integrating imaging, clinical records, genomic data, and patient histories [173]. Tempus applies ML and DL (e.g., CNNs) to clinical and genomic data—including a 100,000-patient database to identify cancer drivers and predict treatment response. It supports personalized therapy, though challenges like data quality, bias, and limited diversity remain [174]. Additionally, model interpretability is an ongoing concern, as clinicians require transparent, actionable outputs to guide patient care decisions.

Flach et al. (2025) [175] explored the integration of Paige Prostate Detect, an AI-assisted tool, into the clinical workflow for prostate cancer (PC) diagnosis. The study aimed to evaluate how AI can improve diagnostic accuracy and efficiency during prostate biopsies. Using deep learning models, including CNNs, Paige Prostate Detect analyzes biopsy slides to identify malignant regions and assist pathologists in detecting areas needing further review.

The system was trained on thousands of annotated biopsy samples [176]. enabling it to assess Gleason scores and distinguish benign from malignant tissues. Preliminary results suggest that AI support may enhance diagnostic speed and accuracy, particularly for less experienced pathologists or challenging cases. However, concerns remain regarding data variability, model interpretability, and the need for large, diverse datasets to ensure generalizability. Importantly, human oversight remains critical to confirm AI-assisted diagnoses.

### AI in drug discovery and repurposing

AI is transforming both patient care paradigms and drug design strategies. Challenges in traditional drug development—such as high costs, time constraints, poor target delivery, and imprecise dosing have prompted the adoption of AI-driven solutions [177]. AI surpasses conventional computational methods by efficiently processing complex datasets, accelerating drug candidate development, and enabling cost-effective solutions. Advanced ML, particularly deep learning, now predicts chemical structures, in vivo/in vitro traits, and outcomes from large datasets, expediting drug discovery without compromising efficacy (Table 6). Platforms like the quadratic phenotypic optimization platform (QPOP) move beyond mechanistic assumptions, tailoring drug combinations to specific disease models or patient profiles using empirical data [178]. AI also enhances patient stratification, drug candidate design, and virtual patient modeling. By leveraging sequencing data like NGS, AI aids in identifying novel therapeutic targets and modeling structure–activity relationships (SARs). Techniques such as ANNs, DNNs, SVMs, GANs, symbolic learning, and meta-learning further optimize drug discovery. The integration of individual patient traits with AI-guided drug prediction is driving a new era of precision medicine, revolutionizing disease management and therapeutic development [179].

**Table 6:** AI in drug discovery and repurposing for cancer therapies

AI Tool / Platform	Application	AI Model Used	Dataset Used	Key Findings / Performance	Clinical Validation Status	Limitations / Challenges	Reference
AlphaFold2	Structure-based Drug Discovery	Deep Learning (Transformer-based)	Protein structure databases	Improved protein structure prediction for drug targets	Used for drug design, integrated with pharma research	Limited by computational cost, accuracy for complex structures	[180]
Atomwise	AI-driven Drug Discovery	CNN-based Model	Molecular datasets	Identified potential drug candidates for leukemia	Preclinical stage	Requires experimental validation, target specificity	[181]
BenevolentAI	AI-based Drug Repurposing	NLP & Graph Neural Networks	Biomedical literature, molecular databases	Identified Baricitinib for COVID-19 repurposing	FDA-approved for COVID-19, extended to cancer studies	Limited labeled data, generalizability issues	[182]
DeepChem	AI in Molecular Modeling	Graph Neural Networks	PubChem, ChEMBL datasets	Enhanced molecular property predictions	Research phase	Needs clinical validation, dataset bias	[183]
Insilico Medicine	AI-driven Drug Discovery	GANs + Reinforcement Learning	Chemical and genomic datasets	Accelerated drug discovery, novel cancer therapies identified	Preclinical validation ongoing	Requires real-world validation	[184]
GENTRL by Insilico	Generative AI for Drug Discovery	Generative Tensorial Reinforcement Learning (GENTRL)	Chemical compound libraries	Discovered novel drug candidates within weeks	Experimental validation ongoing	Scalability, dataset diversity	[185]
Schrödinger AI	AI-assisted Drug Design	ML-based Simulation & Virtual Screening	Molecular Dynamics (MD) datasets	Improved binding affinity predictions for cancer targets	Pharma R&D applications	Requires real-world experimental testing	[186–188]
CANDO (Computational Analysis of Novel Drug Opportunities)	Drug Repurposing & Efficacy Prediction	ML + Network Pharmacology	Drug-target interaction databases	Identified alternative therapeutic candidates for cancer	Research-stage validation	Dataset limitations, prediction accuracy	[189, 190]

*AI* Artificial intelligence, *GANs* Generative adversarial networks, *NLP* Natural language processing, *GNN* Graph neural networks, *GENTRL* Generative tensorial reinforcement learning, *MD* Molecular dynamics, *FDA* Food and Drug Administration, *R&D* Research and development; PubChem, public chemical database, *ChEMBL* Chemical molecules bioactivity database, *CANDO* Computational analysis of novel drug opportunities, *CT* Computed tomography, *MRI* Magnetic resonance imaging, *PET* Positron emission tomography, *SPECT* Single-photon emission computed tomography, *NBI* Narrow-band imaging, *YOLO* You Only Look Once, *TCGA* The Cancer Genome Atlas, *RF* Random forest, *SVM* Support vector machine, *U-Net* U-shaped convolutional neural network

By predicting protein structures at atomic resolution, DeepMind’s DL-based AlphaFold2 has advanced drug development and cancer therapy repurposing. It speed up precision medicine, aids target discovery, and improves understanding of carcinogenic proteins. AlphaFold2 supports structure-based drug design, optimizes ligand interactions, and reveals structures of unresolved proteins [107, 117]. It helps repurpose drugs by identifying new uses, alternate binding sites, and off-target effects. Using ML, it employs transformer-based models, GANs, RNNs, and CNNs. Unlike traditional methods, AlphaFold2 is trained on large datasets from PDB, MSAs, and structural templates, combining spatial and evolutionary constraints via attention-based DNNs. Its accuracy was validated in *CASP13* and *CASP14*, with GDT_TS scores over 85% for medium/difficult proteins outperforming older methods, especially for complex or low-homology proteins. Leveraging HPC resources like GPUs/TPUs, it enables large-scale predictions. AlphaFold2 has modeled critical cancer proteins (e.g., oncogenic kinases, tumor suppressors), aiding inhibitor design and mutation-driven drug development. It also reveals cryptic sites and protein–ligand interactions, enhancing hit-to-lead optimization. Integrated with AI-driven molecular docking, dynamics, and virtual screening, AlphaFold2’s capabilities are further expanded using metagenomic databases, broadening therapeutic target coverage. As a result, it dramatically reduces drug development costs and timelines, enhancing targeted therapy precision.

A study conducted by Guha & Velegol (2023) [181] introduced a DL method incorporating Shannon entropy descriptors to improve molecular property prediction, crucial for cancer drug development and repurposing. Their hybrid DL framework combined GCNs and MLP-based DNNs, with kNN and RF as baselines. Shannon entropy-based features—derived from SMILES, SMARTS, and InChiKey—improved descriptor richness over MW-only inputs. Using 2705 of 3382 data points to predict IC50 (pChEMBL format) for TFPI-targeting compounds, performance metrics included MAE, RMSE, R^2^, and MAPE. Results showed a 25.5% MAPE improvement with entropy descriptors and 56.5% using SMILES-based entropy. DL outperformed kNN, increasing prediction accuracy and reducing experimental costs. Transformer-GNN based DL models have also revolutionized de novo molecule generation, especially for cancer therapies [191, 192]. Companies like Atomwise and BenevolentAI use AI to generate treatment candidates. BenevolentAI applies knowledge graph-based approaches integrating literature, patents, trials, and omics to discover hidden drug-disease links. ML algorithms forecast disease mechanisms, drug-target interactions, and optimal combinations. However, the role of these methods in preventive strategies like chemoprevention remains to be fully explored.

The deep generative models used by Atomwise [193] include transformer-based T5MolGe [194], Mamba, and GPT-based frameworks such as (MolGPT [195], GPT-ROPE [196], and GPT-GEGLU [197]. These models were trained on benchmark datasets such as GuacaMol (1.6 M molecules from ChEMBL 2428) and a carefully selected subset of 171 tyrosine kinase inhibitors (TKIs) from ChEMBL [198]. In addition to goal-directed generation tasks and molecular optimization, the GuacaMol dataset offers evaluation criteria including the created molecules' novelty, uniqueness, and validity. Pytorch, Hugging Face transformers, and Schrödinger Maestro 12.8 for accurate docking were all part of the experimental setup.

Trained on GuacaMol, neural language models like T5MolGe generated 24,700 SMILES for TKIs, filtered down to 7,059 drug-like ligands based on lipophilicity and MWs. DeepPurpose was used for virtual screening, ranking ligands by predicted binding affinities (pKd) to the L858R/T790M/C797S-mutant EGFR in NSCLC. Performance was evaluated using RMSE, MAE, Frechet ChemNet Distance (FCD), and KL divergence. GPT-RoPE had the lowest FCD and highest validity (0.98), while Mamba excelled in FCD and KL, showing effective non-conditional molecule generation. BenevolentAI furthered this approach by integrating biological insights with AI-based hypothesis generation to identify novel therapies and repurpose approved drugs, supporting precision oncology via predictions of drug-protein interactions. While further validation (toxicity, efficacy, pharmacodynamics) is needed, transformer-based models like T5MolGe showed improved conditional generation over traditional DL approaches, supporting cancer therapy repurposing. DeepDDS [199] is a DL model that integrates drug chemical structures and gene expression profiles from cancer cell lines to predict synergistic medication combinations for cancer therapy [182]. Trained on 12,415 drug-pair/cell-line combinations—36 drugs and 31 lines—it used Loewe scores to label pairs as synergistic (> 10) or antagonistic (< 0). DeepDDS outperformed DL and ML baselines in leave-one-out cross-validation and showed a 16% improvement on an AstraZeneca test set. It predicted novel synergies, such as lapatinib with abemaciclib for A375 cells, supported by prior findings in HER2-positive breast cancer [183]. DeepChem provides implementations of related models, though its exact role in DeepDDS is unspecified [182].

De novo drug design [200]. using generative AI/ML avoids template dependency and enhances scalability. RNNs trained on SMILES can learn molecular properties and propose drug-like molecules, though issues like SMILES degeneracy and scaffold similarity persist. To address these, Popova et al. (2018) [201] introduced ReLeaSE, combining generative and predictive RNNs with reinforcement learning (RL) to design Janus kinase 2 inhibitors. However, limitations in SMILES expressiveness led to GNN-based frameworks like DeepGraphMolGen [202] and the Graph Convolutional Policy Network (GCPN) [203], have proven to be more effective than SMILES strings in molecule creation since the emergence of GNNs. In addition to traditional generative methods, de novo drug design has made extensive use of AEs, which are built to learn effective coding of input data, and generative adversarial networks (GANs) [204], which are made up of two neural networks competing with one another to produce new data. To build tiny organic compounds, for instance, Putin et al. (2018) [184] used the Reinforced Adversarial Neural Computer (RANC), which developed unique structures that matched predetermined chemical descriptors while preserving structural integrity. The effectiveness of the GAN framework was improved by later research, resulting in variants like the Adversarial Threshold Neural Computer (ATNC), Molecular GAN (MolGAN), Objective-Reinforced Generative Adversarial Networks (ORGANs), and Objective-Reinforced Generative Adversarial Network for Inverse-Design Chemistry (ORGANIC). Additionally, ChemVAE is the first variational AE based on DL to produce optimal drug-like compounds. Comparably, compounds having specific characteristics, such as a topological polar surface area, partition coefficient (log P), and molecular weight, can be produced by the Conditional Variational AE (VAE; CVAE). The produced compounds showed strong inhibitory effects against targeted disorders, and a hybrid VAE model was developed to build candidates with anticipated potent anticancer actions.

Aggarwal et al. (2021) [185] developed MolGPT, based on GPT-style transformers, generates valid and diverse molecular scaffolds. Advanced models like AAE, LatentGAN, druGAN, and GENTRL further enhance drug design, with GENTRL completing DDR1 inhibitor discovery in 21 days. These AI-driven systems vastly accelerate and de-risk traditional drug development. Y. Li et al. (2023) [205], for instance, used Chemistry42 [206], a well-known platform in this field, to create promising small-molecule inhibitors that target the putative oncogene, CDK8, in order to control advanced solid tumors and acute myeloid leukemia. The most effective chemical exhibited significant antiproliferative effects (IC_50_ = 2.4 nM) and sub-nanomolar enzyme inhibitory activity (IC_50_ = 0.4 nM). These examples demonstrate how data-driven AI molecular generation techniques can create molecules with unique architectures, aiding in the investigation of new therapeutic scaffolds.

To find currently available medications that could be modified to act as RET inhibitors in the treatment of NSCLC, researchers [186] used a thorough computational approach. To predict and evaluate inhibitory effects of possible drugs, the methodology used molecular docking, a density functional theory (DFT) analysis, molecular dynamic simulations, and ML classifiers.

A dataset of 11,808 chemicals from the DrugBank database was used in the investigation. To predict these chemicals' inhibitory activities against the RET protein, ML classifiers were trained [187]. To evaluate their binding affinities to the RET kinase domain, the models' top candidates underwent additional precision docking. To assess the compounds' stability and chemical reactivity, a DFT analysis was performed. Simulations of molecular dynamics were used to comprehend ligand–protein interactions. To guarantee the durability of the connections, molecular dynamic simulations were used to comprehend how the ligand–protein complexes behaved over time. The findings indicated the possibility of a number of drugs as treatment agents for RET-positive NSCLC by highlighting their strong inhibitory effects against RET. Schrödinger [188]. has created sophisticated computational systems that use AI and ML to improve molecular docking and virtual screening, two steps in the drug discovery process. Their method makes it possible to efficiently screen large chemical libraries, which facilitates finding viable medication candidates. Although the aforementioned study did not specifically make use of Schrödinger's tools, the techniques used are consistent with what Schrödinger's software package can perform. Schrödinger's platform, for example, efficiently screens and rescores extremely vast chemical libraries by fusing physics-based techniques with ML-powered active learning. High hit rates across a variety of target classes were demonstrated for this method, indicating its promise for finding new inhibitors for cancer treatments. ​In conclusion, the work serves as an example of how combining computer simulations and ML can make it easier to find possible RET inhibitors for treating NSCLC. The techniques used are representative of cutting-edge computational tools, such those created by Schrödinger, which improve the efficacy and precision of oncology drug discovery procedures.

The Computational Analysis of Novel Drug Opportunities (CANDO) platform finds protein properties that cause a drug’s action and uses ML and Network Pharmacology to enhance drug repurposing. The approach predicts the effectiveness of drugs against a variety of diseases, including cancers, by integrating large-scale proteome-based chemical interaction modeling [189]. The collection includes a thorough drug-protein interaction matrix that compares hundreds of human proteins to thousands of FDA-approved and experimental medications. To find important protein characteristics affecting medication interactions and therapeutic outcomes, CANDO [190] uses proteochemometric modeling with CNNs and RFs to map molecular fingerprints to therapeutic outcomes. Multitarget modeling and feature extraction enhance prediction accuracy, validated via cross-validation and retrospective comparisons with clinical trials (Table 7). The results highlight the impact of protein–ligand interactions on repurposing accuracy and affirm AI's role in advancing multitarget drug discovery in oncology.

**Table 7:** AI in radiation therapy and surgical robotics

AI Solution	Application	AI Model Used	Dataset Used	Performance Benchmark	Clinical Validation Status	Limitations/Challenges	Reference
AI-Driven Cone Beam CT-Based Online Adaptive Radiotherapy	Adaptive radiation therapy	Not specified	Clinical datasets from radiation therapy sessions	Identification of high-risk failure modes; proposed interdisciplinary workflow to mitigate potential errors	Clinical implementation with safety measures	Requires structured workflows to ensure safety and efficiency	[207, 208],
AI-Assisted Augmented Reality Robotic Lung Surgery	AI-assisted robotic surgery in cancer treatment	AI-based approach for augmented reality	Data from robotic lung surgeries	Technological feasibility of AI-based augmented reality in robotic lung surgery	Research phase; feasibility study conducted	Integration of AI with existing surgical workflows; validation in diverse clinical settings	[209–211]
AI-Enhanced Surgical Phase Recognition for Robotic-Assisted Esophagectomy	Improving surgical outcomes and precision medicine	Deep learning models for surgical phase recognition	Video recordings of robotic-assisted esophagectomy procedures	Enhanced accuracy in recognizing surgical phases	Research phase; benchmarking and enhancement studies conducted	Requires large annotated datasets; generalization to different surgical procedures	[212]
AI-Driven Intraoperative Identification of Cancer Metastases	Improving surgical outcomes and precision medicine	Deep learning system for metastasis identification	Intraoperative images from surgeries involving gastrointestinal malignancies	Outperformed oncologic surgeons in identifying peritoneal surface metastases; improved identification by 5% and reduced unnecessary biopsies by 28%	Research phase; requires multi-institutional clinical validation	Integration into existing surgical workflows; validation across diverse patient populations	[213–215]
AI-Enhanced Radiation Therapy Treatment Planning	Dose optimization and adaptive radiation therapy	Artificial intelligence and machine learning models	Data from radiation therapy processes, including imaging and treatment plans	Improved efficiency and accuracy in treatment planning; reduced human intervention	Research phase; studies conducted in low- and middle-income countries	Implementation in resource-constrained settings; need for clinical validation	[216, 217]
AI-Enhanced Surgical Skill Assessment	Improving surgical outcomes and precision medicine	Artificial intelligence models for skill assessment	Surgical video datasets and instrument kinematics data	Automated skills assessments; potential for intraoperative feedback	Research phase; narrative reviews conducted	Development of standardized assessment metrics; validation across surgical specialties	[218, 219]

*CBCT* Cone beam computed tomography, *AR* Augmented reality, *AI* Artificial intelligence, *CT* Computed tomography, *ML* Machine learning, *DL* Deep learning, *RT* Radiation therapy, *EHR* Electronic health record, *WSI* Whole-slide image, *CNN* Convolutional neural network, *GANs* Generative adversarial networks, *NLP* Natural language processing, *GNN* Graph neural networks, *GENTRL* Generative tensorial reinforcement learning, *MD* Molecular dynamics, *FDA* Food and Drug Administration, *R&D* Research and development, *ARS* Augmented reality surgery, *LMIC* Low- and middle-income countries, *KPI* Key performance indicator, *SPM* Surgical phase modeling, *SSA* Surgical skill assessment

### AI in radiation therapy and surgical robotics

**Fig. 5:**
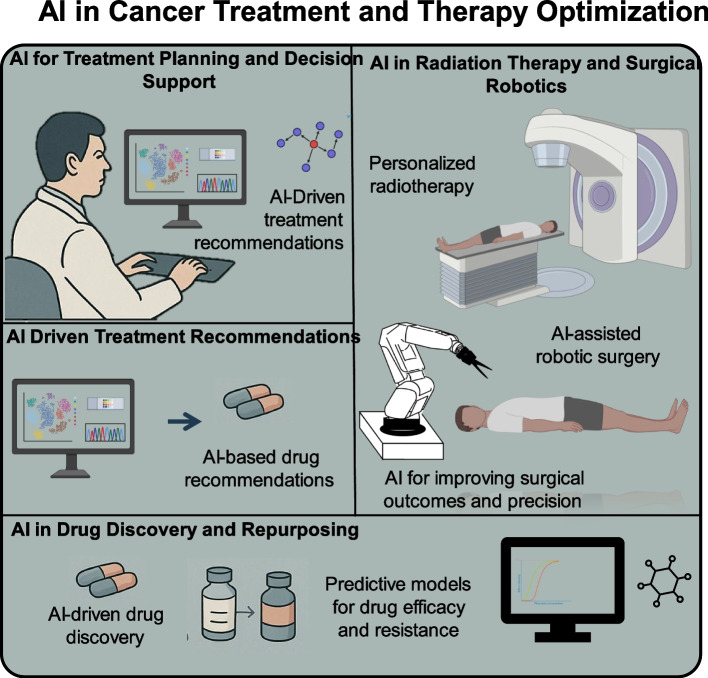
AI applications across cancer care workflows from treatment planning and drug recommendation to robotic surgery and drug discovery. AI enhances decision support, enables personalized radiotherapy, assists in surgery, and predicts drug efficacy and resistance, thereby improving precision, outcomes, and therapy development

Radiation oncology is a kind of cancer treatment that calls for interdisciplinary knowledge from fields such as biology, physics, engineering, and medicine. CT simulations, target registration/contouring, medical imaging, diagnoses, prescriptions, treatment planning, treatment quality assurance, and treatment delivery comprise the standard radiotherapy workflow [220]. The radiation workflow has grown more complex due to technological advancements in recent decades, which have led to a significant reliance on human–machine interactions (Fig. 5). The extensive use of image-guided radiation therapy has produced a vast volume of imaging data that require quick analysis. However, temporal limits restrict people' ability to study and analyze vast amounts of data. However, machines can be trained using AI algorithms to take over many tedious tasks from humans, thereby enhancing the ability to deliver high-quality healthcare.. Many AI-based techniques have been put forth to address issues in many facets of radiotherapy since the advent of DNNs. Given the speed at which AI-assisted radiation is developing, intelligent automation in a number of radiotherapy-related areas could significantly increase the efficacy and efficiency of radiotherapy in the future [58].

#### Surgical robotics and AI: a developing field

With the convergence of surgical techniques and AI, the field of robotics is one that is rapidly developing and has the potential to completely transform surgery. With its capacity for learning, reasoning, and decision-making, AI has the potential to expand surgical robot capabilities (Fig. 5), improving operating room efficiency, safety, and precision [220].

Research demonstrates that collaboration between medical professionals and ML algorithms enhances decision-making and reduces errors. For example, ML-based lung cancer staging reached 93% accuracy, compared to 72% with clinical guidelines alone. By integrating diverse data sources, ML offers more precise and actionable predictions than traditional methods. In surgery, AI improves performance by reducing errors through motion, energy, and force analysis, enabling automated and quantitative skill assessments. Tracking key movement patterns aids in evaluating dexterity, supporting ongoing reevaluation, credentialing, and real-time feedback during training. Ershad et al. [221] proposed evaluating surgical skill by analyzing a surgeon’s "movement style," based on the premise that expert surgeons perform procedures with greater ease, efficiency, and coordination. They collected kinematic data from 14 surgeons of varying experience, each performing two haptic feedback tasks (ring, rail, suture) three times. 3D electromagnetic tracking captured hand, wrist, and shoulder movements during virtual simulations. Training videos were crowdsourced and labeled based on behavioral traits, and a classifier was trained accordingly. This approach improved skill classification accuracy by 68.5% over raw kinematic data. By focusing on qualitative, surgeon-specific motion traits (e.g., smoothness, calmness, synchronization), this method reduces reliance on task-specific surgical knowledge for skill assessment [222].

Cone-beam CT (CBCT)-based online adaptive radiation therapy for postoperative esophageal cancer patients incorporates DL and AI into radiation therapy to improve treatment precision and flexibility [223]. In order to minimize toxicity and enhance treatment results, the study made use of CBCT imaging to track anatomical changes in real time and modify radiation doses accordingly [207]. The dataset included pretreatment and daily CBCT scans of postoperative esophageal cancer patients. Using deformable image registration and DL-based segmentation, AI tracked tumor and organ shifts. Auto-contouring with CNNs and U-Nets accurately delineated targets and critical structures, easing clinician workload. DNNs trained on prior CBCT data enabled effective image-guided radiation adaptation. AI-powered online adaptive radiotherapy improved dose conformity, raised tumor control probability by 10%–15%, and reduced organ-at-risk doses by up to 25%, minimizing complications. These results underscore AI’s potential to personalize and optimize radiotherapy amid anatomical variability [223].

Robotic lung surgery using AI-assisted augmented reality (AR) uses AR and AI to improve thoracic treatments' accuracy, visualization, and decision-making [209]. To improve surgical precision and efficiency, this study employed DL-based segmentation, AI-driven imaging, and 3D visualization. CNNs and transformer models analyzed preoperative CT, MRI, and PET scans to generate detailed 3D reconstructions of lung structures, tumors, and vessels. These models were integrated into AR headsets or robotic consoles for real-time intraoperative guidance. Multi-modal inputs—annotated CTs, surgical videos, and histopathology supported accurate tissue differentiation and effective model training [224].

The study showed that AI-assisted AR lowered the risk of intraoperative complications by improving tumor localization, vascular identification, and margin assessment. According to clinical assessments, the AI-AR system improved resection accuracy by 30% and cut down on operating time by 25% compared to traditional techniques [225]. Additionally, precise robotic-assisted tumor removal, better suturing, and superior postoperative results were made possible by incorporating AI-powered robotic platforms, such as da Vinci surgical systems [226]. AI-driven dose planning in radiation therapy enhances tumor targeting while reducing radiation exposure to nearby healthy tissues. Based on patient-specific imaging data, ML algorithms forecast tumor responses, optimizing radiation regimens for individualized care [210]. Outcomes showed that AI-assisted operations resulted in a 20% decrease in postoperative complications and better recovery of lung function. In thoracic oncology, the integration of AI, AR, and robotic surgery transformed minimally invasive lung cancer treatment, opening the door to safer, more effective, and customized surgical procedures in radiation therapy and robotic-assisted lung surgery [227].

AI-driven intraoperative metastasis detection and surgical robotics integrate deep learning, computer vision, and robotic-assisted surgery to enhance treatment precision. This study explored how real-time AI analysis of intraoperative imaging (fluorescence, hyperspectral, MRI) improved tumor and metastasis identification, guiding surgical decisions and robotic actions [213]. AI models—CNNs, transformers, GANs—processed histopathology, radiology, and intraoperative fluorescence images, refining tumor detection and robotic execution. Trained on over 50,000 annotated histopathology images and imaging scans, these models supported precise, minimally invasive procedures. Systems like the AI-enhanced da Vinci robot use ML-based segmentation and haptic feedback to improve surgical dexterity and minimize tissue damage. AI enables real-time tumor localization using single or multimodal datasets, boosting intraoperative diagnosis and decision support. During Mohs surgery, a DL algorithm created by Sendín-Martín et al. (2022) [214], demonstrated a DL model using ex vivo confocal imaging that rapidly detected basal cell carcinoma with an AUROC of 0.94 in validation. Furthermore, AI algorithms use genetic and radiomic data to predict cancer metastases, constantly modifying surgical techniques to reduce the probability of recurrence. Predictive analytics powered by AI further improve the planning of chemotherapy and radiation therapy, customizing care according to the tumor biology of each patient [215].

Valente et al. [228] explored AI's role in surgical training, focusing on robotic surgery and AI-based skill assessment. AI enhances training by objectively evaluating technique, precision, and decision-making during simulations—areas where traditional assessments often lack consistency. For example, AI can track hand movements, timing, and instrument accuracy in simulated laparoscopic tasks, benchmarking them against expert standards. This enables educators to pinpoint specific strengths and weaknesses. However, AI should complement, not replace, traditional evaluations, as it struggles to assess qualitative skills like clinical judgment and communication.

By learning from surgical movies, AI has been used in surgical robotics to teach robots how to carry out simple surgical procedures. A study team fed a model hundreds of movies taken by da Vinci robot wrist cameras during surgery using ML architectures akin to ChatGPT [217]. This method showed how AI can improve robotic surgical systems and lower medical errors by enabling the robot to lift tissue, stitch, and manage needles. However, current robotic systems can be imprecise and have historically required a lot of coding to teach particular tasks, which can be a time-consuming process. Beyond its application in surgical settings, AI has also significantly transformed other areas of cancer treatment, such as radiation therapy workflow management [229]. For example, a graphical user interface within a commercial treatment planning system, automated template plan preparation, and AI models that predict optimal fluence maps were all features of an in-house AI platform for automated head and neck intensity-modulated radiation therapy (IMRT) [230]. Clinical data were used to validate this system, showing that AI-generated plans reflected their design aims by offering a broad variety of tradeoffs between target volumes and organs-at-risk [218]. In a similar vein, a multistep integrated radiation therapy workflow with AI support was developed for patients with nasopharyngeal cancer, encompassing processes from beam administration to CT scanning. The workflow in a research with 120 patients took a median of 23.2 min, and AI-generated outlines needed only minor adjustments for high-risk clinical target volumes and organs-at-risk. Remarkably, 92.3% of AI-generated designs met dosimetric restrictions for the majority of organs-at-risk following initial optimization. These AI-powered platforms present chances to reduce the effort required by medical personnel, improve the quality of plans, and cut down on treatment planning times. The requirement for thorough validation, possible difficulties integrating with current clinical workflows, and guaranteeing the dependability and security of AI-generated plans in various clinical contexts are some of the drawbacks, though [219].

In contrast to open surgery, AI-based robotics offer numerous benefits by enhancing surgical precision and patient outcomes. Robotic arms provide superior stability and dexterity compared to human hands, allowing for more-accurate surgical techniques. Additionally, robotic-assisted surgery is often less invasive, requiring smaller incisions, which results in reduced trauma, faster recovery times, and improved overall patient well-being [231]. Furthermore, these advanced systems incorporate cutting-edge imaging technologies, such as 3D visualization, which enhance a surgeon’s ability to navigate complex anatomical structures with greater precision and confidence [232].

## AI and patient management in oncology

### AI in prognostic modeling and survival predictions

As genomic data become more standardized and analytical methods advance, AI holds great promise for developing reliable survival prediction algorithms. The complexity and cost of genomic data analysis place a heavy burden on clinicians, making diagnoses and treatment planning dependent on potentially limited expertise. This can lead to delayed or inaccurate decisions. Quantitative, data-driven approaches are thus essential. Advanced ML and DL methods provide targeted solutions, enabling clinicians to enhance treatment planning and improve patient outcomes through innovative learning strategies [233].

A few sequential phases are involved in predicting survival times for cancer patients: (1) preprocessing of genomic data, (2) dimensionality reduction, (3) feature selection, (4) model training, and (5) survival time predictions. A variety of genomic data types, including mRNA, DNA methylation, copy number alterations, and others, are preprocessed during the training phase (Table 8). The model is then trained using a variety of ML approaches after these features either separately or in combination are used to lower the dimensionality [234].

**Table 8:** AI in patient management in oncology

AI Solution	Application	AI Model Used	Dataset Used	Performance Benchmark	Clinical Validation Status	Limitations/Challenges	Reference
MammaPrint	Prognostic modeling and survival prediction	70-gene expression signature	FFPE or fresh tissue samples	Provides binary risk classification for breast cancer recurrence	FDA-cleared; validated in prospective clinical trials	Limited to early-stage breast cancer patients; requires tumor tissue samples	[235, 236]
Continuous Individualized Risk Index (CIRI)	Dynamic risk profiling using serial tumor biomarkers	Bayesian statistical models	Diverse cancer biomarkers over time	Integrates various biomarkers to provide personalized risk estimates	Research phase; further validation needed	Complexity in integrating diverse biomarkers; requires longitudinal data	[237, 238]
Radiomics	AI-based survival analysis and risk stratification	Machine learning models analyzing imaging features	CT, MRI, PET scans	Identifies imaging biomarkers associated with prognosis and treatment response	Research phase; ongoing studies	Variability in imaging protocols; need for standardization	[239–241]
EvieAI	AI-powered wearable device for cancer patient monitoring	AI chatbot trained on medical journals	Data from wearable devices	Provides accurate health information with 99% accuracy	Beta version available to Evie Ring users	Ensuring user privacy; avoiding diagnostic advice	[242]

*FFPE* Formalin-fixed paraffin-embedded, *FDA* Food and Drug Administration, *CIRI* Continuous Individualized Risk Index, AI Artificial intelligence, *CT* Computed tomography, *MRI* Magnetic resonance imaging, *PET* Positron emission tomography, *ML* Machine learning, *DL* Deep learning, *NLP* Natural language processing, *GNN* Graph neural networks, *Bayesian* Bayesian statistical models, *R&D* Research and development, *WSI* Whole-slide image

Using layered ANNs in conjunction with supervised or unsupervised learning approaches, sophisticated DL algorithms automatically integrate feature selection, dimensionality reduction, and prediction into a single procedure. DL models typically outperform conventional ML techniques in forecasting survival times because they aim to uncover hidden patterns and relationships. DL is becoming increasingly well-liked as a potent technique for genomic data analysis due to the growing availability of genetic data and sophisticated processing capabilities [243].

Current research being published frequently uses clinical or imaging data to forecast survival times for cancer patients. These approaches, however, might not always yield precise forecasts and do not make full use of the abundance of information found in genetic data. There are not many reviews on survival predictions of cancer patients using genomic data, they are not particularly thorough, and there are no comparisons of various ML models that can inspire future studies [244].

#### ML techniques for predicting cancer survival

Including genetic data enhances survival time prediction but introduces high dimensionality, requiring careful consideration. To address this, researchers often apply dimensionality reduction or feature selection techniques. Survival prediction models typically consist of two components: the predictive model and dimensionality reduction. These methods fall into two categories—supervised and unsupervised—with common approaches including principal component analysis (PCA), factor analysis, and non-negative matrix factorization (NMF) [245].

It is possible to reduce features using dimensionality reduction or feature selection techniques. Furthermore, new paradigms like the Multi-Cancer Multi-Omics Clinical Dataset Laboratories (MCMOCL) [246] schemes, which use federated learning, AE, and XGBoost techniques to improve accuracy, that decrease processing delays and improve security in heterogeneous cancer clinics, have been introduced by recent developments in digital healthcare. Other studies have investigated hybrid cancer detection schemes that use State-Action-Reward-State-Action (SARSA) reinforcement learning [247] and multi-omics data processing in fog cloud networks with the goal of improving accuracy and decreasing processing times in distributed clinical settings [248]. ML enables accurate survival prediction from complex genomic data, using methods like SVM, AdaBoost, RF, and decision trees. Key considerations include feature selection to reduce noise, interpretability for clinical relevance, and validation (e.g., cross-/external validation) to ensure model robustness [249]. ML-based cancer survival prediction faces challenges like data heterogeneity, bias, overfitting, and poor interpretability. Small, imbalanced datasets reduce generalizability. Addressing these requires advanced feature engineering and regularization to ensure clinical reliability [250].

### AI in remote monitoring and digital health

Digital health, commonly known as "eHealth" or "healthtech," represents the convergence of technology and healthcare, and its importance in oncology is immense. In the realm of oncology, digital health involves a wide range of technologies, strategies, and innovations aimed at enhancing cancer prevention, diagnosis, treatment, and management through digital means [251]. These technological solutions include, but are not restricted to, electronic health records (EHRs), mobile health applications, wearable technology, telemedicine services, and analytics powered by AI. It has the potential to completely transform oncology by providing cutting-edge solutions that improve patient care, diagnoses, and treatments. With developments in AI, VR, AR, predictive analytics, international cooperation, and changing legislation, the future of digital health in oncology appears bright [252]. Digital health enables early cancer detection through large-scale analysis of genetics, lifestyle, and imaging data, supporting timely, personalized treatment. By tailoring therapies to individual genetic profiles, it improves outcomes and reduces side effects [253].

Digital health tools like wearables and telemedicine enable remote monitoring, improving quality of life for cancer patients and reducing hospital visits. The data collected accelerates research, drug development, and treatment optimization [254]. As a catalyst for innovation, digital health holds the potential to transform global cancer care and significantly improve patient outcomes.

### The Role of digital health in enhancing oncology outcomes

#### Enhanced diagnostics and personalized treatment

Integrating digital health technologies, including genomic sequencing and liquid biopsies, has significantly advanced cancer diagnostics by facilitating early detection. These innovative tools enable the identification of malignancies at their earliest stages, often before clinical symptoms become apparent. Early detection is of paramount importance as it substantially increases the probability of successful therapeutic outcomes. By diagnosing cancer at an incipient stage, clinicians can implement timely and targeted treatment strategies, improving survival rates and enhancing patients' overall quality of life [255].

#### Optimized care coordination and personalized treatment approaches

Digital health plays a crucial role in developing individualized cancer treatment plans by leveraging patient-specific genetic and molecular data. Through advanced analytics, healthcare providers can tailor therapeutic interventions to align with the unique genetic profile of each patient and the molecular characteristics of their malignancy. This precision-based approach enables the administration of treatments that directly target the biological mechanisms underlying the disease, thereby maximizing therapeutic efficacy while minimizing adverse effects [256].

#### Patient empowerment and enhanced care coordination

Integrating EHRs serves as a foundational element in optimizing care coordination within oncology. EHRs consolidate comprehensive patient data, including one’s medical history, diagnoses, treatment regimens, prescribed medications, and laboratory results. This centralized information repository ensures that all healthcare providers involved in a patient's care, including primary care physicians, oncologists, radiologists, and other specialists, have immediate access to up-to-date medical records. Consequently, streamlined communication and collaboration among multidisciplinary teams reduce the likelihood of medical errors and facilitate more-informed clinical decision-making [257].

#### Telemedicine and remote consultations

Virtual healthcare technologies, including telemedicine, have become essential in improving care coordination in oncology. These digital solutions enable remote consultations, allowing oncologists and specialists to collaborate irrespective of geographical constraints. By fostering seamless interdisciplinary communication, telemedicine facilitates timely expert consultations, expediting diagnoses and treatment initiation. Ultimately, these advancements ensure that cancer patients receive prompt, specialized care while mitigating delays associated with geographical and logistical barriers [258].

### AI for enhancing clinical trials and patient recruitment

Clinical trials remain central to safe and effective drug development. With the rise of data-driven and personalized medicine, it's essential for companies and regulators to adopt tailored AI solutions that enhance research speed and efficiency. AI is increasingly recognized for its potential to support sustainable and optimized drug development, with several applications now being explored. To streamline drug research, robust AI models trained on appropriate datasets are needed to extract actionable insights, especially as data availability grows in personalized healthcare [259].

Clinical trial enrichment focuses on selecting patient subsets where drug effects are more evident, rather than testing efficacy in a general population. Including non-responsive patients can dilute observed outcomes. Ideally, genome-to-exposome profiling would guide eligibility by confirming relevant biomarkers, though such trials are rare and costly—particularly when imaging is involved. Thus, biomarker testing should be applied wherever feasible, even without full omics profiles. To uncover actionable biomarkers and subpopulations, advanced analytics must integrate omics with fragmented data from EMRs, imaging, and handwritten notes. Tools like NLP, OCR, and computer vision automate this extraction. Yet, the volume and inconsistency of EMR data complicate analysis. AI models, being data-agnostic, are well-suited to harmonize these inputs, supporting trial enrichment and biomarker discovery—though care is needed to prevent overfitting, especially with class imbalance [260]. At least four molecularly different forms of breast cancer have been identified through gene-expression profiling investigations [235]. Several genetic tests have been developed to better predict clinical outcomes and assess if the addition of adjuvant chemotherapy to endocrine therapy is worthwhile. Cardoso et al. (2016) [261] assessed the clinical utility of the MammaPrint 70-gene expression signature for guiding adjuvant chemotherapy in early-stage breast cancer. By stratifying patients into low- or high-risk groups based on recurrence-associated gene expression, MammaPrint offers genomic insights beyond standard clinical-pathological criteria to reduce unnecessary chemotherapy. The phase 3 trial included 6,693 women, with genomic risk determined via MammaPrint and clinical risk via a modified Adjuvant! Online tool. Patients with matching risk profiles (low-low or high-high) were treated accordingly, while discordant cases were randomized. Among 1,550 women with high clinical but low genomic risk, the 5-year survival rate without chemotherapy was 94.7% (95% CI: 92.5%–96.2%), with only a 1.5 percentage point drop in distant metastasis-free survival compared to those who received chemotherapy. The study found that chemotherapy use could be reduced by 46.2% in high clinical-risk patients when guided by MammaPrint. This supports its role in enabling more personalized treatment and reducing overtreatment and side effects for low-genomic-risk patients. Still, challenges include limited long-term data, cost and accessibility barriers, and difficulty integrating genomic testing into routine care. The modest decline in survival also emphasizes the importance of careful patient selection and shared decision-making. In parallel, Kurtz et al. (2019) introduced the Continuous Individualized Risk Index (CIRI), a Bayesian model aimed at improving outcome prediction in chronic lymphocytic leukemia (CLL) patients undergoing targeted therapy [238]. The CIRI model improved therapy response predictions by continuously combining a number of prognostic markers, both clinical and molecular, while responding to changes in a patient's state.

The methodology uses a Bayesian data analysis to simulate the link between different risk variables and progression-free survival (PFS). The CLL International Prognostic Index (CLL-IPI) considers age, immunoglobulin heavy chain (IGHV) mutation status, TP53 mutation or deletion, staging information (Binet or Rai) [262], and serum β2-microglobulin levels. There are also minimal residual disease (MRD) levels mentioned, which offer information about how well a treatment is working. Because the Bayesian system continuously updates predictions based on changing patient data throughout therapy, it enables dynamic risk assessments.

Clinical trial data from 699 patients in the CLL8, CLL10, and CLL11 studies were among the datasets utilized to create the CIRI-CLL model. With a 23%–30% improvement in the C-statistic for predicting PFS over 1–5 years, the data demonstrated that CIRI performed better than conventional risk models, such as the CLL-IPI. CIRI successfully categorized patients into different risk groups, which were connected with various 3-year PFS rates (77.1% for low-risk, 54.5% for intermediate-risk, and 9.4% for high-risk patients), according to validation using the CLL14 trial cohort (432 patients) [263]. The CIRI model offers a number of advantages, especially when it comes to customizing treatment regimens. By more precisely predicting which patients may benefit from particular medications, it enables clinicians to tailor their recommendations based on each patient's unique risk profile, increasing results [264]. Additionally, it facilitates continuous, customized patient risk monitoring as their condition changes. Its dependence on clinical trial data, which might not accurately reflect the overall population of CLL patients, and the requirement for additional long-term validation to evaluate its efficacy in standard clinical practice are drawbacks. Furthermore, there can be difficulties incorporating this model into current clinical workflows, especially in environments with limited resources. Avanzo et al. [239] examined radiomics and AI in cancer imaging, highlighting how ML and DL models (e.g., RFs, SVMs, CNNs) extract and analyze quantitative features to improve diagnosis and prognosis. DL, particularly CNNs, outperformed traditional ML in tasks like tumor segmentation and classification, using datasets like TCIA [240]. However, challenges remain, including the need for standardized imaging protocols, larger datasets for DL training, and validation across diverse patient populations. Despite these limitations, the integration of ML and DL in radiomics presents significant opportunities for more accurate, personalized medical predictions, advancing precision medicine in oncology and beyond.

Bakshi B et al,. [241] evaluated the impact of C the Signs, an AI-powered clinical decision support tool, on cancer diagnosis rates in primary care. The observational cohort study included nearly 420,000 patients across 35 practices in eastern England (May 1, 2021 – March 31, 2022). The platform analyzed comprehensive patient data—including medical history, tests, treatments, medications, demographics, and risk factors—using AI to assess cancer risk and recommend diagnostics or referrals. Practices using C the Signs saw cancer detection rates (CDRs) rise from 58.7% (2020–2021) to 66.0% (2021–2022), a 12.3% increase (*p* < 0.05), while non-using practices maintained a stable CDR of 58.4%. Notably, referral rates remained similar between groups, suggesting improved detection did not lead to over-referral. The findings underscore the potential of AI tools in primary care to enhance early cancer detection and enable timely intervention, potentially reducing cancer-related mortality.

Movano created the AI chatbot EvieAI, which is included into their Evie Ring, a smart ring with a wellness and health theme [242]. EvieAI, which was unveiled at CES 2025, stands apart for being post-trained solely on more than 100,000 peer-reviewed medical papers written by medical experts. Because it cites data from reputable sources like the Mayo Clinic, Harvard, and UCLA before answering, this method guarantees that the information it offers is accurate and reliable. By comparing its answers to various reliable sources, Movano asserted that EvieAI attained a 99% accuracy rate.

EvieAI is a conversational resource that focuses on women's health and seeks to assist users by providing answers to questions about wellness and health without making diagnostic recommendations. It is designed to recognize when it lacks an answer and refrains from responding to non-medical questions. For example, when a user exhibits symptoms, EvieAI might probe more deeply to gain a better understanding of the situation, but for more-serious problems, it will refer users to the proper resources or medical experts. The design of EvieAI places a strong emphasis on security and privacy. In order to protect user confidentiality, the platform uses industry-standard encryption for data transfer and storage, guarantees that discussions stay anonymous, and periodically removes conversation data.

## Challenges and limitations of AI in cancer care

The benefits for cancer treatments still appear far off, despite the fact that AI applications in oncology continue to hold enormous promise. There are still many significant issues and concerns, such as the difficulty of standardizing, gathering, and managing data; the bias present in training datasets; the absence of strong reporting guidelines; the relative dearth of prospective clinical validation studies; difficulties implementing user designs and workflows; antiquated legal and regulatory frameworks surrounding AI; and the exponential growth of knowledge and dynamic data.

### Collection and administration burdens associated with data standardization

Unstructured, unique, and diverse methods are frequently used to record and retain healthcare data. As a result, AI algorithms created using data from one system might not work as effectively when used with data from another system. For AI to significantly influence oncology, the percentage of EHR data that is ontology-integrated will rise as a result of standardizing nomenclature and data gathering. These issues are being addressed by initiatives like the minimal Common Oncology Data Elements (mCODE) initiative, but it will require a lot of work to widely and reliably implement solutions [265].

### Prejudiced training data

Pattern recognition is the main emphasis of AI as it exists now. As a result, any pattern seen in the data used to create the model will be carried over into predictions that the model produces. There being consistent differences between the data used to construct the model and the data to which the model is applied could be an issue [266]. For instance, traditionally underrepresented populations (such as women, ethnic minorities, adolescents and young adults, and the elderly) in a dataset may have an impact on AI's capacity to produce an accurate recommendation for these specific subgroups when clinical trial data are used as the basis for the algorithm [266]. To avoid this kind of bias, it is also critical to guarantee representative sampling across time (for example, recently treated versus previously treated patients) and data sources (for example, medical record data from various health systems). It can be concluded that inherent biases that are frequently found in training datasets will need to be addressed by AI-based solutions [267]. Computational techniques are being developed to identify, comprehend, and reduce prior bias in training datasets. Potential remedies might include creating techniques to quantify the bias of a given data collection and defining criteria that define when bias is severe enough to raise doubts about using that dataset as a target for deployment or for training algorithms [268].

### Absence of prospective clinical validation and research reporting guidelines

The lack of reporting guidelines for AI has led to a reproducibility crisis, which may prevent AI from being widely used. Lack of repeatability is a serious concern that could be challenging to overcome because AI systems, particularly DL techniques, are sensitive to minute details in data that cannot be discovered. This issue might be resolved by tightening reporting regulations on source codes and training circumstances of algorithms, but openness might also cause issues with intellectual property and competitive advantages for businesses that use AI [269]. While the broader background of AI studies in healthcare remains without common use reporting standards, some specialties have started formal guidelines. For example, for radiology, the CLAIM (Checklist for Artificial Intelligence in Medical Imaging) [270] guideline is an all-encompassing framework to provide transparency in study design, data handling, model development, evaluation, and clinical deployment. Complementing these organization-specific efforts, the FUTURE-AI [271] framework, developed by 117 experts from 50 countries, outlines 30 best practices for trustworthy AI in healthcare, based on six principles: fairness, universality, traceability, usability, robustness, and explainability. It promotes standardized, safe, and clinician-ready AI across the full development lifecycle, emphasizing the need for domain-specific and cross-domain frameworks.

### Challenges with workflows and user designs

**Fig. 6:**
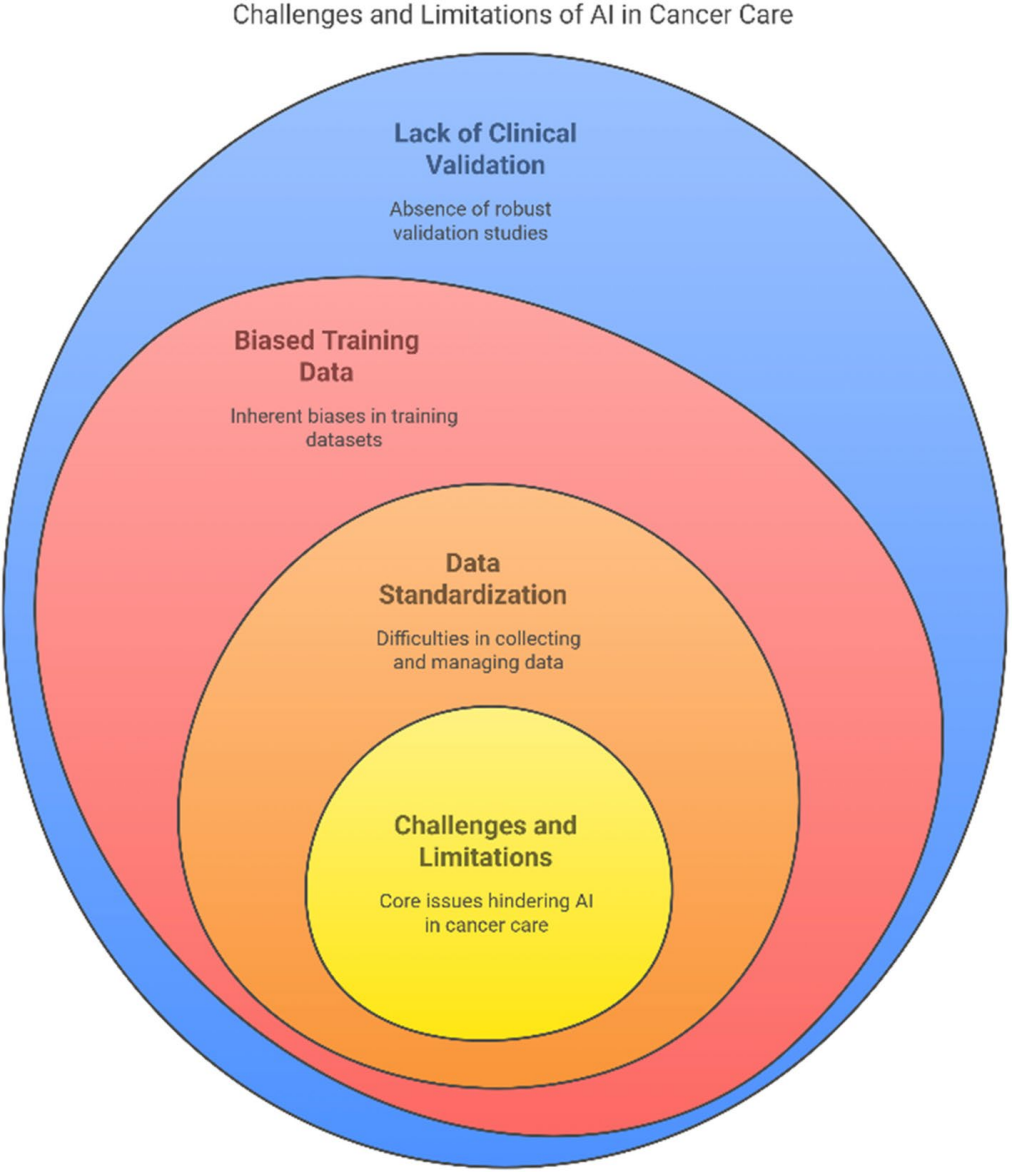
Key challenges limiting AI adoption in cancer care: core issues include lack of data standardization, biased training data, and insufficient clinical validation. These nested problems collectively hinder the reliability, generalizability, and clinical utility of AI-driven cancer diagnostics and treatments

The sociotechnical issues that arise in intricate adaptive healthcare systems must be addressed for AI to be successfully implemented. AI-based solutions must be easy to use, add value for the user, and blend in smoothly with a clinician's workflow in order to promote broad adoption. This is a bigger obstacle for some AI applications than for others [272]. Key elements for adoption include having output that is both explainable and actionable, as well as being seamlessly integrated into clinical processes, even though not all AI systems that analyze data must be made available to doctors through interactive interfaces. However, oncologists' clinical decision support systems (CDSSs) frequently need more engaging and informative interfaces [273]. The end-user must be able to see the dynamic features of AI-based solutions to the degree that they are multidimensional or adaptive. For instance, the physician must be able to see these aspects if AI-based CDSSs adapt over time to changes that take place during therapy (Fig. 6) (such as anatomical and physiological changes to the tumor and surrounding normal tissues during radiation) [273].

### Dynamic data and knowledge

Algorithms used in real-world oncology scenarios will need to keep up with the exponential rise in cancer research regardless of the regulatory framework in place. Additionally, they will need to take into consideration dynamic changes in source data that may be brought about by new diagnostic technologies, upgrading of EHR systems, expansion of data standards and ontologies, or shifts in documentation and reimbursement rules. It is necessary to develop techniques to periodically assess algorithm accuracy or change algorithms when their performance begins to deteriorate due to changes in underlying data distributions. It might also be necessary for some algorithms to incorporate an automatic expiration feature, which would force reevaluation after a predetermined amount of time [274].

## Future perspectives and emerging AI trends in oncology

**Fig. 7:**
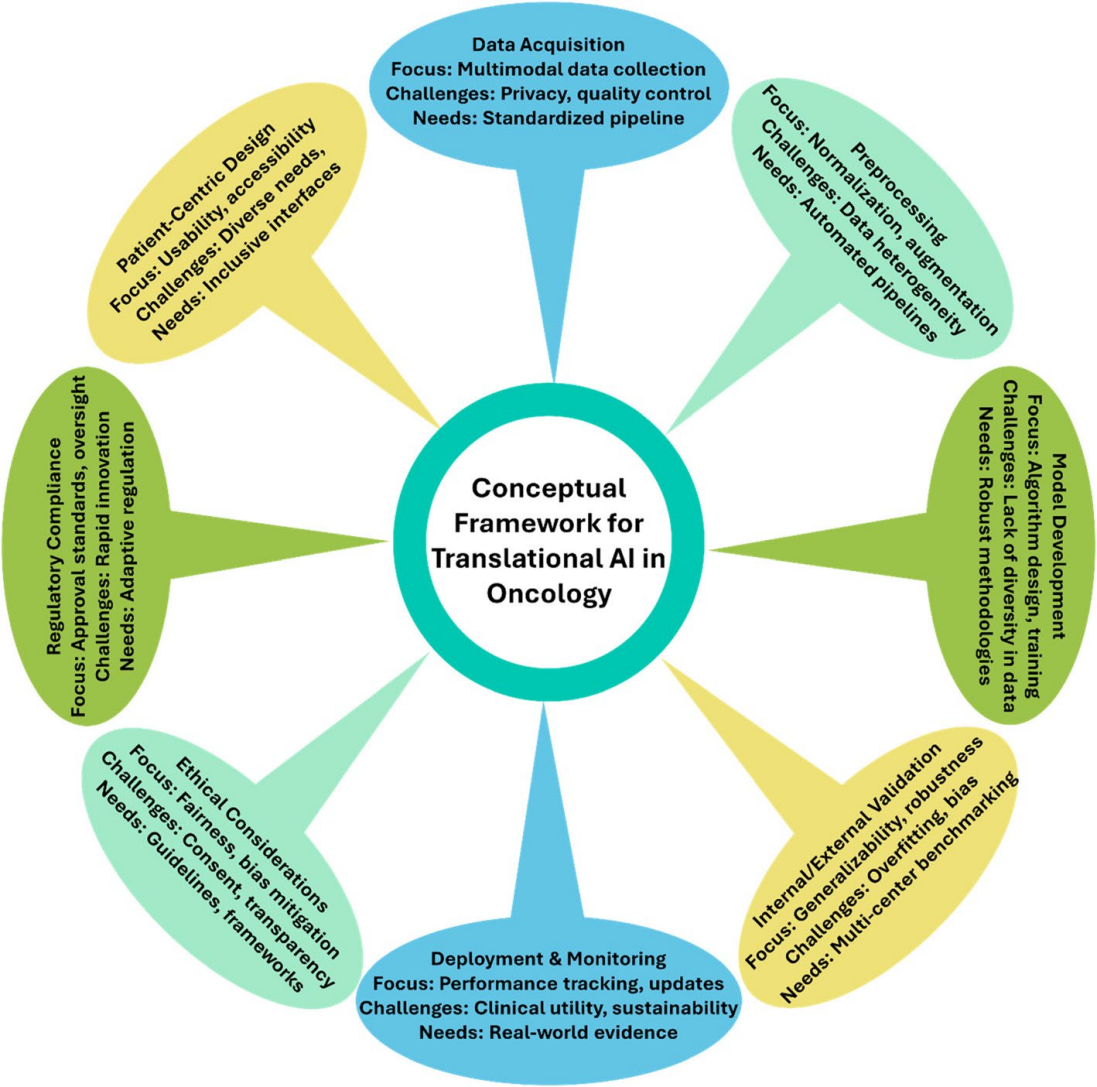
Eight-point conceptual framework for translational AI in oncology. This framework delineates eight critical areas required for effective AI deployment in cancer therapy: Data Acquisition, Preprocessing, Model Development, Internal/External Validation, Deployment & Monitoring, Ethical Considerations, Regulatory Compliance, and Patient-Centric Design. Each one is defined by its prime purpose, primary challenges, and strategic needs. All these dependent factors make up an end-to-end handbook for AI development toward safe, ethical, and equitable clinical release in oncology

Thanks to advancements in big data analytics, AI, and customized treatments, the healthcare industry is poised for dramatic changes in the near future. These developments have enormous potential to optimize healthcare delivery, and improve patient care and health outcomes. However, in order to guarantee responsible and equitable deployment, their incorporation into healthcare systems also presents difficult ethical conundrums that need to be carefully handled. This section looks at the state of healthcare going forward, highlighting the significance of cooperation between different stakeholders, the influence of emerging technologies, and ethical issues. In order to combine the multidisciplinary progress and issues in AI oncology, we present an integrated conceptual framework (Fig. 7). This framework identifies eight key domains that are essential for taking AI from research to practice with clinical efficacy, encompassing the entire pipeline from data acquisition to post-deployment and ethics.

**Fig. 8:**
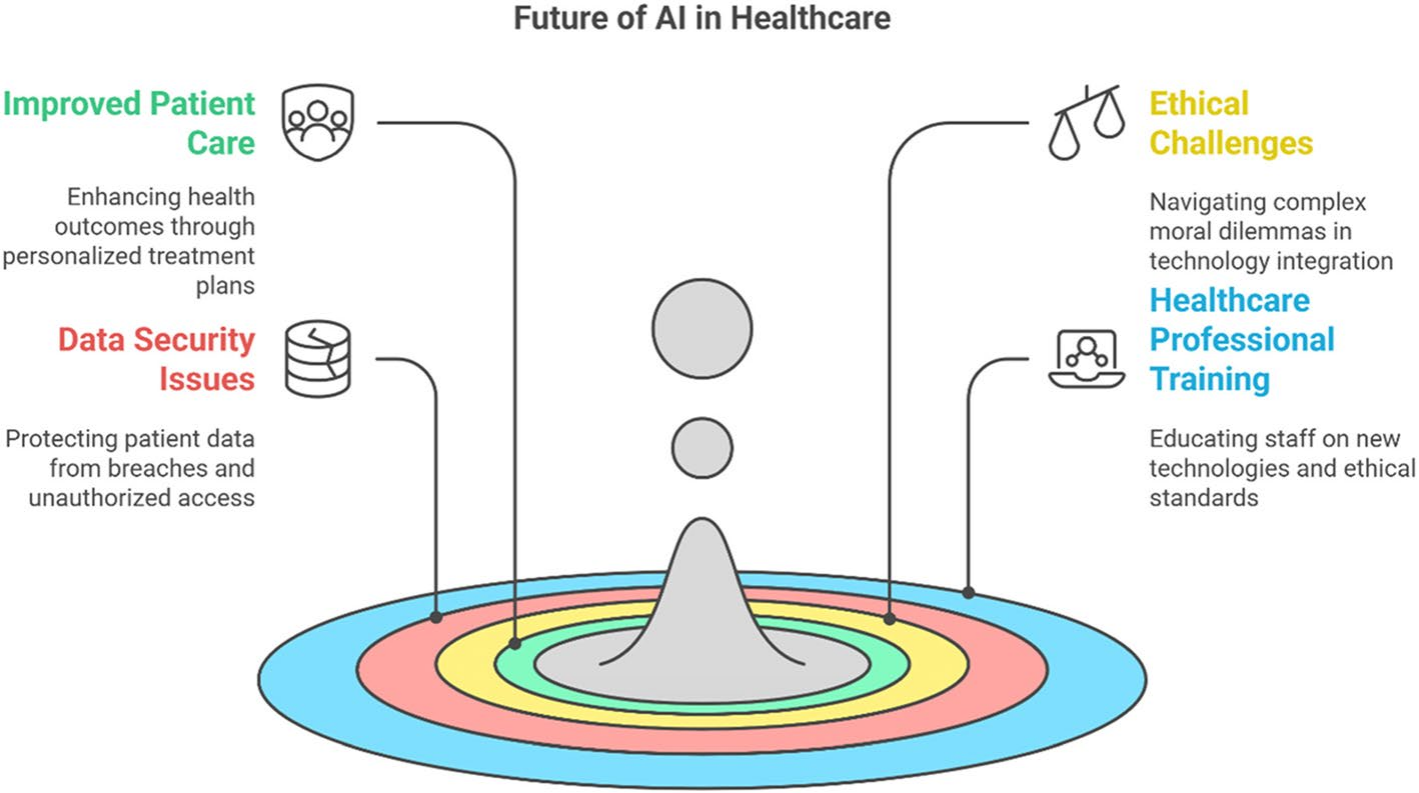
The future landscape of AI in healthcare, focusing on four key domains: improving patient care through personalization, safeguarding data privacy, addressing ethical dilemmas in AI use, and training healthcare professionals to effectively implement and govern new technologies with responsibility and skill

AI-driven innovations are poised to transform diagnosis and treatment, with algorithms already enhancing medical imaging, predicting disease progression, and personalizing care. AI has demonstrated superior early detection capabilities, particularly in cancer, and is expected to further improve diagnostic precision and individualized treatment as it evolves. Big data analytics also holds promise for advancing healthcare by uncovering insights into disease prevention, treatment efficacy, and population health trends. However, the use of large-scale patient data raises concerns about privacy and security. Robust data protection measures and legal frameworks emphasizing patient consent are essential to ensure individuals retain control over their data (Fig. 8). As AI, big data, and personalized medicine continue to grow, healthcare professionals must be trained not only in technical skills but also in ethical considerations. Education should focus on best practices for integrating these technologies into care while upholding ethical standards. By fostering a culture of ethical awareness, healthcare systems can responsibly leverage technology to improve patient outcomes [275].

## Conclusions

AI is no longer a secondary adjunct in oncology—it is becoming an essential, intrinsic component in advancing cancer therapeutics. By seamlessly integrating heterogeneous biomedical datasets into clinically actionable insights, AI is transforming every stage of cancer care: detection, diagnosis, treatment, follow-up, and research. This review underscores both the vast promise and complexity of embedding AI into oncology, spanning imaging modalities (CT, MRI, PET, ultrasound), histopathology, genomics, proteomics, and more. AI shifts clinical decision-making from subjective estimations to high-accuracy, algorithmic diagnostics that often outperform conventional methods in speed, reproducibility, and precision. Beyond diagnosis, AI enables personalized treatment planning, fine-tuned radiation dosing, enhanced robot-assisted surgeries, and discovery of novel therapeutic targets via data-intensive drug development pipelines. On the patient management front, AI-powered wearables and virtual assistants facilitate real-time remote monitoring, boost treatment adherence, and detect complications early. In clinical research, AI optimizes study design, patient stratification, and recruitment through real-time eligibility checks. Yet despite these advancements, challenges remain in achieving universal clinical adoption. Concerns about algorithm transparency, reproducibility, and interpretability underscore the need to build trust among providers and patients. Regulatory frameworks for AI in healthcare are still evolving, and comprehensive governance models ensuring safety, efficacy, and innovation are urgently needed. Critical data-related challenges—bias, inequity, security, and interoperability—must be addressed, particularly as biased training data risks exacerbating existing health disparities across demographics and regions. A multidisciplinary ecosystem—uniting AI researchers, oncologists, ethicists, regulators, and patient advocates—is essential to create equitable, transparent, and clinically valuable AI deployment standards. Medical education must evolve to equip future healthcare professionals with the skills to responsibly apply AI in clinical practice. Looking forward, AI’s convergence with federated learning, edge computing, digital twins, and quantum ML offers exciting potential for highly granular, scalable, and personalized cancer care. Emerging synergies between AI, synthetic biology, and de novo immunotherapy design point toward truly individualized next-generation treatments. Ultimately, deploying transparent, privacy-preserving, and ethics-focused AI models will foster trusted healthcare systems. AI’s true potential lies not just in improving current practices but in reshaping oncology into a predictive, preventive, participatory, and precision-driven discipline. With a human-centered approach and collaborative innovation, AI can usher in a transformative era in cancer care—benefiting all patients through smarter data use, outcome-driven strategies, and inclusive clinical impact.

## Data Availability

No datasets were generated or analysed during the current study.
